# Lightweight Proof of Game (LPoG): A Proof of Work (PoW)’s Extended Lightweight Consensus Algorithm for Wearable Kidneys

**DOI:** 10.3390/s20102868

**Published:** 2020-05-19

**Authors:** Adarsh Kumar, Deepak Kumar Sharma, Anand Nayyar, Saurabh Singh, Byungun Yoon

**Affiliations:** 1 Department of Systemics, School of Computer Science, University of Petroleum and Energy Studies, Dehradun 248007, India; adarsh.kumar@ddn.upes.ac.in; 2 Department of Informatics, School of Computer Science, University of Petroleum and Energy Studies, Dehradun 248007, India; dksharma@ddn.upes.ac.in; 3 Graduate School, Duy Tan University, Da Nang 550000, Vietnam; anandnayyar@duytan.edu.vn; 4 Faculty of Information Technology, Duy Tan University, Da Nang 550000, Vietnam; 5 Department of Industrial & Systems Engineering, Dongguk University, Seoul 04620, Korea; Saurabh89@dongguk.edu

**Keywords:** game theory, blockchain, cryptocurrency, lightweightness, gash rate, bit-exchange, challenge-response, attacks, healthcare

## Abstract

In healthcare, interoperability is widely adopted in the case of cross-departmental or specialization cases. As the human body demands multiple specialized and cross-disciplined medical experiments, interoperability of business entities like different departments, different specializations, the involvement of legal and government monitoring issues etc. are not sufficient to reduce the active medical cases. A patient-centric system with high capability to collect, retrieve, store or exchange data is the demand for present and future times. Such data-centric health processes would bring automated patient medication, or patient self-driven trusted and high satisfaction capabilities. However, data-centric processes are having a huge set of challenges such as security, technology, governance, adoption, deployment, integration etc. This work has explored the feasibility to integrate resource-constrained devices-based wearable kidney systems in the Industry 4.0 network and facilitates data collection, liquidity, storage, retrieval and exchange systems. Thereafter, a Healthcare 4.0 processes-based wearable kidney system is proposed that is having the blockchain technology advantages. Further, game theory-based consensus algorithms are proposed for resource-constrained devices in the kidney system. The overall system design would bring an example for the transition from the specialization or departmental-centric approach to data and patient-centric approach that would bring more transparency, trust and healthy practices in the healthcare sector. Results show a variation of 0.10 million GH/s to 0.18 million GH/s hash rate for the proposed approach. The chances of a majority attack in the proposed scheme are statistically proved to be minimum. Further Average Packet Delivery Rate (ADPR) lies between 95% to 97%, approximately, without the presence of outliers. In the presence of outliers, network performance decreases below 80% APDR (to a minimum of 41.3%) and this indicates that there are outliers present in the network. Simulation results show that the Average Throughput (AT) value lies between 120 Kbps to 250 Kbps.

## 1. Introduction

**Figure 1:**
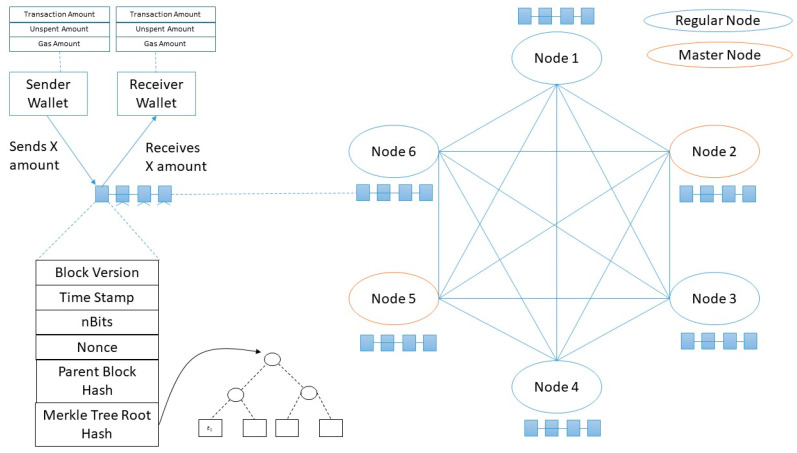
Blockchain and Its Network.

Blockchain is a peer-to-peer distributed data-ledger network constructed using consensus among nodes in a network as shown in Figure 1. Blockchain networks can be permission or permission-less. Blocks in blockchain network (BN) stores verified transaction records and are connected with relevance to its predecessor. The header of each block contains a cryptographic challenge and its solution. Nodes (miners) interested in adding a block to existing BN has to solve this challenge (usually in 10 min) before getting permission to add a block. This cryptographic challenge is generated using various means that are meaningful in consensus building [1,2] in different applications.There are various applications of blockchain in different domains. Some of these applications are as follows:

- *Banking:* Blockchain technology can replace both commercial and investment banking. Blockchain technology has solutions to every service of banking like international money transfer, letters of credit, forex, escrow, custody, initial public offering, mergers and acquisitions, restructuring, trading etc. In the banking system, people deposit their money and banks give them credits. Nowadays, banks charge a huge amount of their services. These charges can be minimized and services can be made faster with the help of a blockchain network. Further, a large part of the population is not associated with the banking system due to numerous reasons and blockchain can bring them into mainstream banking which would be a win-win situation for both investor and economy. Blockchain can control inflation thus it is useful to countries having high inflation rates. Overall, it is very helpful in almost every national or international money-related transactions. Batavia [3] is an example of a blockchain-based trade finance platform to tackle inefficient processes in the business. It is developed using the IBM blockchain platform and consists of five banks (UBS, Bank of Montreal (BMO), CaixaBank, Commerzbank and Erste Group) and IBM. This platform aims to remove inefficient cross-border trading barriers. For example, all paper-based activities (bills, letters of credits etc.) are maintained electronically, money can be paid at different stages, tracking of goods exchange is much easier etc. Nordea has a blockchain-based platform as well to ease the trading finance for SMEs in Europe.
- *Insurance:* Blockchain is peer-to-peer based technology and it is helpful in insurance section by reducing the uncertainty in business, back-office costs and bureaucracy work is also reduced, faster execution of processes as compared to traditional ways, customer relationships and their satisfaction is improved with the usage of electronic smart contracts and their execution etc.
- *Healthcare:* This is an important sector where the necessity of blockchain is highly required. This sector demands accurate, immutable and easily accessible patient’s historical and present medical records. In healthcare, industry 4.0 processes are required alongside blockchain technology. Industry 4.0 will be the communication backbone of the healthcare system, whereas, blockchain technology builds trust in the data-related system (collection, exchange, retrieval storage etc.). In healthcare, blockchaincan be used starting from patient records, drug supply system, staff’s records, medical insurances, medical trials, equipment usage and supply system, medical board approval system, government monitoring system etc.
- *Intellectual Property Rights (IPRs):* It is an important area where the importance of blockchain technology is realized in recent times. Using blockchain, IPR’s ownership, copyright protection, and smart IP rights management are managed efficiently. This is possible through distributed ledger technology in the blockchain. For example, IP rights are managed from the moment of their inception rather than the time of patent or publication. This protects the invention and false claims. IPRs using blockchainare helpful in various domains like software, music, films, research, business etc. For example, payments can be made instantaneously for the usage of anything (watching a mobile, listening to music etc.) and it is possible for even a microsecond. As blockchain is a P2P system using smart contracts thus it is efficient, secure, faster and economical.
- *Food Supply Chain:* There are many departments associated with the food supply business as well. For example production, processing, distribution, marketing, and consumption. In real scenarios, present supply chain management is not trustworthy because of various incidents like illegal practices, frauds, improper food storage systems, laborious and error-prone data records, for example.

Likewise, there are many other areas such as supply management system, transportation sector, smart city, industrial networks, etc., where blockchain technology can bring a great revolution through data transparency, immutability, security, availability etc. As the blockchain technology is widely accepted in several domains, the challenges in adopting this technology in a systematic, secure and trustworthy way are also very large. Various important challenges in blockchain network-based applications are briefly explained as follows:

- *Scalability*: In blockchain networks, every node has to communicate transaction information to every other node in the network. With the increase in the number of nodes in a network, the number of transactions increases exponentially. In a blockchain network, every node does not have the same resources/configuration. This restricts the feasibility of extending the Blockchain network beyond a certain size.
- *Restricted Blockchain Users*: One major application of blockchain is to bring those people in the banking domain who live in rural areas or do not have any banking solutions. However, the limited blockchain network’s scalability restricts these users to become part of the blockchain-based banking system. As the world population is increasing day-by-day, requirements for a large scale blockchain-based applications are increasing proportionately. Otherwise, it would restrict the usage of applications to certain people only.
- *Resource limitations*: Various concepts in the blockchain network consumes large resources. For example, an electricity-based PoW consensus algorithm permits nodes to add several blocks proportionate to electricity consumed or produced. In both cases, the chances of building a large blockchain network reduces. Other popular cryptocurrencies are based on a platform that uses a proof-of-stake (PoS) consensus algorithm. Although various attempts have been made in implementing PoW and PoS integrated consensus solutions, no pure PoS-based blockchain solution has been implemented yet in a real application.

In healthcare, various challenges include scalability and performance, usability, interoperability, and adoption. These challenges are briefly explained as follows:

- A large amount of data is generated from medical records including electronic health records or electronic medical records [4,5,6,7]. Blockchain features makes the data to be stored at every node with hash values. This will increase the storage demands and requirements.
- Patient data should be easy to access as and when required. Manual or partial automated data access does not solve the problem. A complete medical record with required statistics is mandatory for a doctor before treatment. Similar importance is required to all form of data exchanged in the healthcare domain whether it is for drugs, suppliers, producers, suppliers, government policies etc.
- Interoperability is another major challenge. In the present scenario, healthcare organizations are targeting cross-discipline or specialization interoperability for patient treatment rather than patient-centric approaches. A patient-centric approach is more transparent, useful and secure.
- Adoption is another major challenge in healthcare. Many sub-systems in healthcare are used for unethical and economic gains. This would bring down the importance of healthcare. Thus, there is a need to design more transparent and efficient mechanisms.
- Performance is another major issue. In populated countries or pandemic situations, a large number of patient approach hospitals. Thus, an exponentially large amount of data is processed which lower down the overall system, processes.

After understanding the importance of blockchain-technology, healthcare systems, and Industry 4.0 processes, this work considers a wearable kidney-based system for data collection, sharing, processing, and analysis. Wearable kidneys have many electronic and sensor components and these components can be controlled remotely through advanced processes for medication and handling during emergency cases. Here, blockchain technology can be integrated to obtain records of kidney functionalities in the form of transactions. Here, the individual component is generating the block and they are integrated to construct the blockchain. The successful construction of blockchain ensures that kidney record is successfully recorded.The exchange of information to a remote location is ensured through Industry 4 processes. These processes allow data analysis, interpretation, secure exchange, and provide timely information to patients and doctors as well. Such records are helpful for analysis later and subsequent medications. While keeping all importances of blockchain into consideration, the objectives designed for this work are briefly explained as follows:

- To integrate Healthcare 4.0 processes with a patient-centric healthcare system. Healthcare 4.0 includes Industry 4.0 processes (Internet of Things (IoT), Industrial IoT (IIoT), cognitive computing, machine learning, AI, etc.) integrated with the healthcare system. Healthcare 4.0 processes are capable to handle a large amount of data efficiently. The major aim of this integrate is to design a large scale patient-centric healthcare system and test the performance.
- To consider a wearable kidney patient system and simulate it with Healthcare 4.0 processes. A wearable kidney consists of electronic, medical, fluid, and other systems. The integration of all of these components with an automated consensus algorithm ensures smooth functionality. Thus, a lightweight consensus algorithm is integrated. Further, Healthcare 4.0 processes ensure faster data accessibility and statistical results.
- To proposed lightweight and new challenges-based consensus algorithms and integrated with the wearable-kidney patient system and determines the performance. As a consensus algorithm is part of the blockchain network, a blockchain-based consensus algorithm with game theory is proposed for healthcare. The proposed algorithm is variable in bits-based challenge generation and verification. The performance of the blockchain network with a lightweight consensus algorithm is required to be determined as well.
- To consider different patients with kidney diseases and simulate it with a change in the amount of data required to treat them. Further, the performance of these cases is required to be determined for analyzing the proposed framework efficiency.

In this work, the healthcare domain (specifically the kidneys) are selected for sensor-integrated information network construction. This work has started with the design and analysis of normal kidney operations, its parts, and the possibilities of diseases. Thereafter, a model is designed and analyzed for a wearable kidney system. This wearable system design is integrated into a novel IoT, IIoT, cloud and cognitive computing-based model. This system is designed to monitor (doctor and self-monitor by a patient) the working of medical and electronic equipment based wearable kidney system. Further, the proposed system is integrated with Industry 4.0 processes and to have Healthcare 4.0 processes for managing patients on a large scale. To integrate blockchain technology-based advantages in the proposed system, the PoG-based consensus algorithm is proposed for resource-constrained wearable kidney devices and applicable to both permission and the permissionless blockchain networks. Here, three PoG-based consensus algorithms are proposed: Single-Player Single-Bit (SPSB), Multi-Player Single-Bit (MPSB) and Multi-Player Multi-Bits (MPMB). Thereafter, these algorithms are integrated into the wearable kidney-based Industry 4.0 network. Finally, the performance of these consensus algorithms is evaluated and compared to identify the best algorithm for resource-constrained devices.

The rest of the paper is organized as follows: Section 2 explores the state-of-the-art survey related to the consensus algorithms and healthcare systems. Section 3 presents the normal and wearable kidney systems. This section also shows the proposed integration of kidney systems with Industry 4.0 processes. Further, proof-of-game (PoG) algorithms are proposed in this section that is used in overall kidney based information networks to have blockchain technology advantages. Section 4 shows the simulation of the proposed system, results and analysis. Finally, Section 5 concludes the work.

## 2. Background and Related Work

This section has explored the state-of-the-art consensus algorithms, and existing blockchain-based systems or applications in healthcare. Various cryptographic challenge generation approaches are as follows [1,2]:

- *Proof-of-Work (PoW):* Nodes in BN put forward a challenge and a miner solves the challenge for verification. Resource consumption in generating a solution to a challenge could be one parameter selected for PoW.
- *Proof-of-Authority (PoA):* In PoA, those nodes are given permissions to generate a new block that has proven their authority through preliminary authentication.
- *Proof-of-Elapsed Time (PoET):* This concept is similar to PoW but concentrates more on-time consumption rather than resource utilization.
- *Proof-of-Stake (PoS):* In this concept, the node’s stake is cross-checked before considering it for validation. The amount of stake spend in BN is another possible scenario for considering it for the validation process. This encourages more and more spending to become a validator.
- *Delegated Proof of Stake (DPoS):* This concept is similar to PoS but give more chance for voting and selecting a validator which is having comparatively more stake than any other node.
- *Leased Proof of Stake (LPoS):* In this concept, nodes are having the flexibility to customize tokens to enhance the security.
- *Proof of Importance (PoI):* In PoI, the importance of node increases with its transactional activities which include net amount transfer, amount to which node is closely associated, network transactional activities etc. As each transaction record is immutable in the blockchainnetwork thus PoI overcomes the loopholes of PoS or PoW in which dishonest behavior can increase or decrease the node’s importance and chances to add a block.
- *Proof of Capacity (PoC):* In PoC, a node’s available hard drive space is used to decide the right to add a block rather than its computing power. Computing a power-based PoW consensus algorithm is not useful because every device can have different computing power or device usage at a different time which can result in a different outcome, whereas, PoC counts the availability of space. A larger space means more memory is available to write the problem and its solution. Aim of this consensus algorithm is that if more memory is available to write all possible solutions to a problem then chances of success increase. Thus, PoC is a better solution as compared to PoW. This approach enforces the node to efficiently utilize the memory space.
- *Proof of Burn (PoB):* Stewart invented PoB analogous to PoW without energy waste. In PoB, nodes burn or destroy the virtual currency tokens for getting the right to add a block proportionate to coins burnt. In other words, nodes burnt coins to buy a virtual mining rig for getting the power to add blocks. An increase in the number of burnt coins increases the chances to buy mining rig which in-turns increase the chance to add a block.
- *Proof of Weight (PoWeight):* Like PoS, PoWeight assigns ‘weight’ to nodes based on how much cryptocurrency is held on the network. Unlike PoS, weights can be assigned based on different values. For example, a higher node’s weight value indicates a proportionally large amount of stored data. Large weights for thins indicates another consensus system like proof-of-reputation (PoR). In the PoWeight, the Algorand consensus model is the preferred consensus algorithm that constructs a secure and decentralized public ledger based on pure proof of stake rather than proof of work [2].

Additionally, various other challenge generation or consensus algorithms are possible such as Practical Byzantine Fault Tolerance (PBFT), Simplified Byzantine Fault Tolerance (SBFT), Delegated Byzantine Fault Tolerance (DBFT), Directed Acyclic Graph (DAG), etc. The major challenge in these consensus algorithms is to generate a new challenge every time because the old challenge is disclosed and it can be considered secure for a blockchain network. Further, the applicability of these cryptographically based challenge generation approaches varies from application to application.

The algorithms, systems, and applications are explained as follows:

Mingxiao et al. [8] reviewed the principles and characteristics of the consensus algorithms, measured the performance, and identify the application areas/domains. In this work, a technical sequence of steps is derived to guide the selection of a consensus algorithm in a specific domain. Further, limitations and future directions are presented for the consensus algorithm in the blockchain technology. This systematic review explores the core blockchain-technology and research aspects. However, detailed research aspects need to be explored further in detail.

Smith et al. [9] identified the features of a sequence mining platform that includes a machine, storage medium, and sequence manager. Here, a sequence manager is programmed to use processing resources in determining the sequence of nucleobases in nucleic acid. In the storage manager, data is collected from one or more transactions using proof of work. Secondly, some of the data is inserted in the new block of a transaction. To have proof of work for the newly created block, a sequence mining module is required that determines the sequence of nucleobases. In all of these transactions, a rewarding system is integrated that takes care of the transaction and sequencing point rewards. Overall, it is observed in this work that proof of work is an ideal way of establishing the consensus among blockchain nodes.

Azbeg et al. [10] presented an IoT and blockchain-based approach for monitoring diabetes, its follow-ups, patient self-management approach, patient data collection procedure, healthcare data, security in data privacy etc. This work realizes the importance of sharing real-time data with health entities in the smart healthcare system. The proposed system collects the data and shares it with concerned entities. Further, it quantitatively and qualitatively measured the importance of sharing the data in this work. In the proposed system, it is realized that proof of authority is the best approach for consensus establishment because it is faster and economical in terms of saving time and energy. It is also realized that proof of concept can give similar results. However, the experimentation with this consensus approach is not yet implemented and tested. There are equally likely chances of amelioration of two consensus algorithms to achieve similar results.

Kalogeropoulos [11] presented a system that can be used in multiple areas. For example, the proposed system can be used in detecting chronic kidney symptoms with the help of machine learning algorithms and medical records. This approach can be applied over a secure container that can securely receive the health condition predictions by ensuring a privacy-preserving strategy. Other applications of the proposed approach include forecasting the power generation of photovoltaic cells, household energy consumptions, off-chain resource-intensive computations that can monitor or control on-chain values. In chronic kidney disease prediction scenarios, various stakeholders considered are laboratory, container provider, and research institute. A laboratory has the role to prepare patients’ health databases, keep this information secure and private, use application software to store in on-chain network values, take the support of selected containers, perform computations as per the payments and give accurate, appropriate and timely results. On the other hand, the research institute deals mainly with the dataset through software and store the required details. The container contains the sample data and usage fees and the container provider stakeholder receive the fee for storing the required details in containers.

Swan [12] proposed a disease causality in global health care equivalency as a theoretical model using data science concepts. The idea of proposing a smart health network in this work is to design a framework having secure and automated advanced medical and healthcare technologies like medical nanorobots. Medical nanorobots are nanodevices that can be used for maintaining and protecting the human body parts against pathogens. In this work, blockchain technology is used to have immutable and transparent medical records collected through medical nanorobots. Here, bio-crypto-economy and personal biological directed activities (bioDAC) terms are coined. Bio-crypto-economy is used to have crypto-economic principles and issues discussed and applied in biological domains. Whereas, the personal bioDAC is the complete set of programs necessary to understand the purpose-directed biological activities.

Table 1 shows the comparative analysis of other blockchain-based approaches in healthcare systems. In recent work that uses blockchain technology in the healthcare domain has various concerns such as [8,9,10,11,13,14,15,16,17,18,19,20]: (i) there is no system being proposed who take care of artificial medical equipment through a decentralized, secure and efficient way, (ii) the majority of the blockchain-based healthcare systems are theoretical frameworks or limited to detailed designs. However, very few examples are available who have taken care of the verification and validation of proposed processes through statistical or implementation approaches, (iii) most of the consensus algorithms suggest proof-of-work as a leading consensus algorithm in blockchain-based healthcare applications. However, proof-of-work is found to have various implementation challenges, especially for resource-constrained devices. The medical wearable devices (e.g., kidney) required quick responses to have better control thus lightweight and efficient approaches are required to build consensus with improved performance, (iv) applicability of game-theory based challenge in consensus building is not explored much and it can provide a dynamic challenge-based environment to have a better and timely control system, (v) Industry 4.0 processes are adding security features to enhance the scalability and accessibility in industrial processes. Thus, Industry 4.0 processes and blockchain technology-based healthcare system is required to be proposed for improved results, (vi) the existing approaches are not sufficient to have a detailed output result in metric analytics-based verification and validation model. Thus, there is a strong need to build a statistical or implementation based model to measure the real-time performances and QoS, and (vii) the application of deep learning and neural network with blockchain technology are found in the literature. This approach can give more automates patient’s feature matching results. Thus, this area needs to be researched in a systematic and detailed manner.

## 3. Proposed Approach

This section explains the kidney models (generic and wearable) designed in JaamSim [38]. These models are required to be integrated with Healthcare 4.0 processes in the futuristic smart healthcare system. Thus, the Healthcare 4.0 processes-based model is presented. Further, this section explains the proposed consensus algorithms for wearable kidney operations. Here, multiple models with the statistical approach are presented to have efficient and optimized performances. The detailed functionalities of two major categories of proposed approaches are presented as follows:

### 3.1. Proposed Kidney Models

This section explains the generic and wearable kidney processes, their needs, models, and operations in detail. Further, the processes of integrating the kidney model with Healthcare 4.0 processes are explained.

#### 3.1.1. Generic Kidney Model

**Figure 2:**
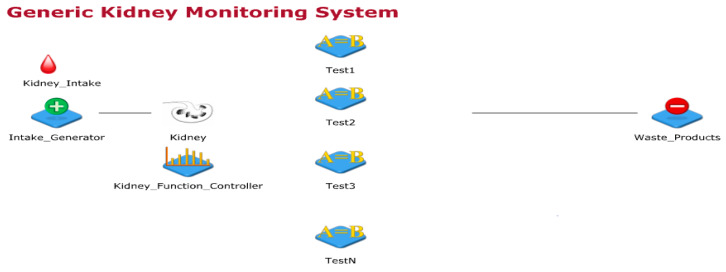
Generic Kidney Monitoring System.

The kidney is an important part of the human body, it works day and night to clean blood and form urine by filtering water and waste from it. Thus, urine travels from kidney, bladder and then out from urethra. Further, the kidney keeps the body’s fluid balance, electrolyte levels, urine waste removal, blood pressure regulation and maintain red blood cells (RBCs) counts. Few of the initial kidney abnormal functions include pain in urination or back-side under the ribs, vomits, nausea, high fever etc. These signs and symptoms vary with age. Kidney infections can be life-threating if proper and timely medical care is not provided. A generic kidney model designed in JaamSimis shown in Figure 2. In this sub-section. this work started with generic kidney processes explanations, designing the basic kidney process model, designing the wearable kidney model, presenting the need and design of the internet of things based wearable kidney model, and presenting healthcare 4.0 based kidney model processes.

*Generic Kidney Processes*: The following are the basic kidney processes in urine formation from plasma:

- *Filtration*: A normal kidney filters around 180 L of fluid every day and a plasma volume is around 3 L. Overall, the complete plasma volume is filtered 60 times per day using hydraulic pressure in the cluster of nerves, spores or small blood vessels importantly known as capillaries of around kidney tubule also known as the glomerulus.
- *Reabsorption*: Also known as tubular reabsorption is a process in which nephron removes water and solutes from pre-urine fluid (tubule) and return into the plasma. As the water and solutes are absorbed once and it is returned back thus it is called reabsorption. In the reabsorption process, around 70 L of the filtered water and solute are returned. In the loop of Henle, sodium reabsorption occurs as well in bulk.
- *Regulated Reabsorption*: In this process, hormones monitor and estimates the systemic conditions and act to control the rate of water and sodium in the distal tubule and collecting duct.
- *Secretion*: This process enhances the kidney’s ability by removing certain wastes and toxins into tubular fluid. This is considered to be an essential process in regulating potassium concentrations and pH values.
- *Excretion*: This is the outcome of the three processes filtration, absorption, and secretion. Here, initial tubule fluid concentration of a substance is similar to plasma but in subsequent reabsorption and/or section it differs in the urine. In other words, if $E_{amount}$, $F_{amount}$, $R_{amount}$ and $S_{amount}$ represents the excretion filtered, reabsorbing and secreted amounts then all can be co-related using:
  (1) $$E_{amount}=F_{amount}-R_{amount}+S_{amount}$$

*Kidney Function Tests*: The kidneys are a vital part of the human body as they filter waste products from the blood and expels from the human body through urine. The kidney controls various levels of water, necessary minerals and critical in producing vitamin D, red blood cells and hormones for regulating blood pressure. There are simple blood and urine tests that can be performed over regular intervals to identify the issues with your kidneys. These tests are classified into urine, blood, imaging and kidney biopsy [39].

*Urine Tests:* These are important tests for a kidney because it is related to waste outcomes of the kidney. An expert doctor recommends having these tests regularly for monitoring the kidney abnormalities. Various tests in this category include urinalysis, urine protein, microalbuminuria, and creatinine clearance test. These tests are briefly explained as follows.

- *Urinalysis:* This process is used to verify the presence of protein and blood in the urine. A protein may occur in the body due to various reasons like infection, heavy physical activities etc. To measure the products of muscle tissue (called creatinine), urine samples are collected over a 24-h collection. This regular measurement indicates the formation of creatinine clearing from a human body.
- *Urine Protein:* This test is usually performed in urinalysis. However, a separate dipstick can be used to find proteinuria (excessive protein). Here, a specific dipstick test which includes albumin specific dipstick or albumin-to-creatinine ratio can be used to have quantitative dipstick test measurements.
- *Microalbuminuria*: This test is recommended by doctors to those patients who have diabetes or high blood pressure issues. In this test, a tiny amount of protein (albumin) is identified in urine samples. After standard dipstick test finding as negative, this more sensitive dipstick test is performed.
- *Creatinine Clearance Test*: In this test, the amount of waste product produces in the kidney per minute is measured. This measurement is performed by a comparison of the creatinine in the 24-h sample to the creatinine level in the patient’s blood. Additionally, this test indicated normal muscle wear and tear in the body.

*Blood Tests:* Like urine tests, blood tests are equally important. These tests help to measure protein, nitrogen or kidney filtering rate. Various tests in this category include serum creatinine, blood urea nitrogen (BUN), and estimated glomerular filtration rate (GFR). These tests are briefly explained as follows.

- *Serum Creatinine*: This is a blood test that measures the creatinine level in the human body. According to [39], signs of kidney problem starts if creatinine level is higher than 1.2 milligrams/deciliter (mg/dL) for women and 1.4 mg/dL for men.
- *BUN*: This test measures the amount of nitrogen in the blood. Nitrogen is a breakdown product of protein present in the human body and consumption of common medications, antibiotics can also result in higher BUN value. Thus, it is very important to regularly measure and maintain this record for doctor recommendations. A normal BUN reading varies between 7 to 20 mg/dL and higher than this could indicate certain other health issues.
- *GFR:* This is a very important test for a patient having kidney disease and it measures the kidney wellness with a measurement of how good is the kidney in removing the wastes and excess fluids from the blood. As a kidney have multiple filtering units (called nephrons), GFR is the sum of all functional nephrons’ filtering rate and it can be calculated indirectly from patient’s age as:
  (2) $$GFR=140-Patient^{\prime }age$$

There are different formulas in GFR computations. Two of these formulas are important and frequently discussed include Cockcroft & Gault, and the Modification of Diet in Renal Disease (MDRD) [40]. The Cockcroft & Gault formula is:

(3) $$\begin{array}{l} CreatinineClearance\\ =\frac{\left[140-Patient^{\prime }ageinyears\right]\times LeanBodyWeight\left(LBW\right)\times 1.23\;\left(formein\right)mls/min}{serumcreatinine\left(umol/L\right)} \end{array}$$

(4) $$LBW=Patient^{\prime }heightincentimeter-100$$

Here, the LBW value is the same as of serum creatinine value if the patient is a woman and kidney function is normal. In a real scenario, if a patient’s age is lesser than LBW value than the actual weight is used for measurements. MDRD GFR calculation formula is:

(5) $$\begin{array}{l} GFR\;\left(mL/\min per\;1.73\;m^{2};1.21\right)\\ \;=186.3\times serumcreatinine\left(e^{-1.54}\right)\times Age\left(e^{-0.203}\right)\times A\times B \end{array}$$

Here, *A* = 0.742 if the patient is female and *B* = 1.21 if the patient is african-american. It is also necessary to observe that GFR decreases with age. In normal range, the kidney is considered to be fit if its GFR ranges from 100 to 140 mls/min. It can have mild kidney failure symptoms if GFR value is less than 90 mls/min. A moderate failure if it goes below 60 mls/min. A severe kidney failure, if it is below 30 mls/min and kidney, is at end-stage if GFR value is below 15 mls/min. A value below 15 mls/min strongly indicates the complete failure and necessity of either any type of dialysis or transplantation.

*Imaging Tests*: Using image-based tests (like ultrasound or CT scan), kidney structural abnormalities or vein obstructions are observed through image-based proofs. Various tests in this category re-explained as follows.

- *Ultrasound:* In this test sound waves are passed to get the kidney’s picture. This test helps in finding the kidney abnormalities (both in terms of size or position). This test is frequently used in finding the kidney stones or tumors that are getting common in most of the patients.
- *CT Scan*: This is another imaging test that uses X-ray technique for getting the kidney’s picture. Like ultrasound, this test is also useful in finding the obstructions (like kidney stones) or structural abnormalities. This test uses intravenous contrast dye inserted into the kidney for getting the image. This dye can be harmful to those that are already facing kidney diseases or abnormalities.

*Kidney Biopsy:* In this test, thin needles with sharp cutting edges are used to slice kidney tissues. Small pieces of kidney tissues are examined with a microscope for various reasons like identification of a disease or its categories, identification of kidney stage i.e., how much kidney is damaged or if some treatment is undergone then is it progressive or create harmful effects over the kidney, finding the cause of kidney transplantation failure etc.

*Test-performing criteria*: Various kidney function tests either require a small or continuous sample. Majorly urine and blood samples are collected. A brief explanation to test performing criteria is explained as follows.

- *Urine Sample*: There are various criteria to perform a urine test. Some may require a very small amount of urine only whereas others require a full 24 h samples. If there is a need to measure the quantity of urine produced then 24-h samples are required. On the other hand, a set of tests to determine protein, blood, sugar etc. require a small sample only.
- *Blood Samples*: In blood-based tests, a small quantity of blood sample is collected by slipping a hollow needle into the patient’s skin and vein. The sample collected is sent to the lab under proper monitoring of an expert doctor and a detailed report is prepared with a unique patient identification mechanism.

Figure 2 shows the generic kidney monitoring system model designed using the JaamSim simulator. In this simulation-model, inputs to the kidney are artificially planned with random kidney controlling functions. Additionally, a simulated environment of kidney function tests is designed which either collects samples from the kidney (considered as the human body part) or from urine. Input to imaging tests and biopsy are randomly provided through the kidney controller. Presently, the simulator model is not mapped to real data for experimental comparisons but this model considers random inputs and gives unpredictable outputs. This way of experimentation is performed to have unknown cases and their remedies.

#### 3.1.2. Lightweight and Wearable Kidney Model

**Figure 3:**
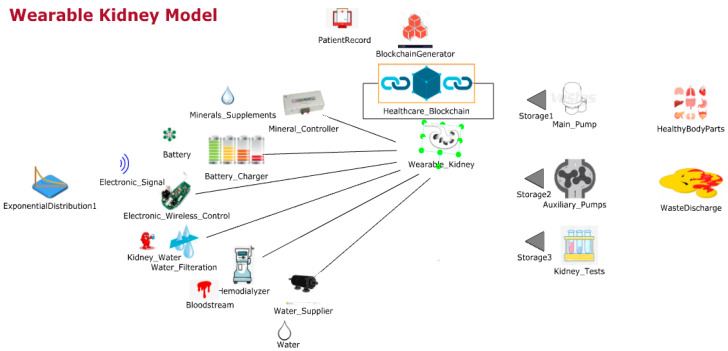
Wearable kidney monitoring system with blockchain technology.

Figure 3 shows the JaamSim model for wearable kidneys with blockchain technology. The wearable kidney monitoring system model is different from the generic kidney model in many terms. In real-scenarios, it has been observed that kidney dialysis processes are very expensive and not very successful for quality of life [41,42]. 

Thus, there is a need for solutions with a high success rate and patient satisfaction. In recent times, the wearable kidney is designed and successfully experimented with over three patients as well [42]. However, it is presently not available to the public because still two studies/experiments are going on to have a much higher success rate in the wearable kidney’s dialysis process. The designed wearable kidney is lightweight, easy to cover under cloths, better control over blood pressure, lesser pressure over the heart by reducing the fluid weight, reduces the medications and have much lesser diet restrictions [42]. In [43], a comparison of five artificial kidney models is presented. Presently, one wearable artificial kidney model is designed for hemodialysis and three are designed for peritoneal dialysis process. The fifth nanodialysis model (named the NaNo) is designed to process both hemodialysis and peritoneal dialysis processes. Figure 3 shows the simulated model for wearable kidneys designed in present times. Various components of the wearable kidney model are briefly explained as follows.

- *Mineral Controller:* This system controls the quantity and quality of minerals (compound, creatinine, uric acid, sodium, amino acid, sugar, protein, nitrogen, blood, water etc.) required for a kidney.
- *Battery System:* Wearable kidneys are known to be lightweight as well and one of the reasons for this is the use of light batteries. These batteries can be charged overnight with fewer resources [42]. This system gives power to every electronic circuit and equipment to operate and control the activities.
- *Electronic Wireless Control:* This is an electronic circuit that is wearable artificial kidney but it is considered to be wireless in our proposed model because to enhance the data collection feature for blockchain network and IoT-enabled platforms. Further, this would add additional individual technology benefits to the overall model. Thus, in addition to a patient monitoring system (planned for present wearable kidney models), the proposed model has data collection and sharing features.
- *Water Filtration:* This system applies various filters and membranes to do impure water filtration and regeneration. This is considered to be the copy process of generic kidney mapped in a simulated environment. The pumping system is also considered to be a sub-system of this system.
- *Hemodialyzer or peritoneal dialysis:* This system is designed to have a dialysis process. In the simulation, it is considered to have success dialysis process and results in irrespective of its type. Dialysis membrane and dialysate regeneration are considered to be part of this system only.
- *Water Supplier:* This system is a source of external, fresh and purified water supply to an artificial kidney system.

#### 3.1.3. Wearable Kidney Model with Edge Computing and Internet of Things Network

**Figure 4:**
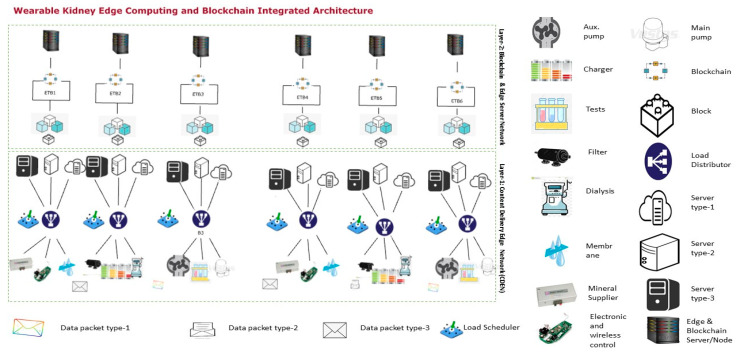
Proposed wearable kidney edge computing with IoT and blockchain technology.

Figure 4 shows the JaamSim model for wearable kidneys attached to a local internet of things network with edge and cognitive services suitable for family or local hospital services. This model is divided into two layers (layer-1 and layer-2). In layer-1, this model collects the wearable kidney data electronically or through a wireless medium. The collected data is classified and shared uniformly among all content delivery network (CDN) servers. These servers are simulated to provide quick service response with high processing time and minimum delay. In processing, communication and computational speed complexities are computed, whereas, delay counts processing, transmission and propagation delays. Thereafter, blockchain and edge server processing are applied in layer-2. In a blockchain network, blockchains are constructed for patient medical records (patient ID, blood pressure, glucose, lactate, bicarbonate, calcium, sodium, urea, amino acid, creatinine, urine appearance, ketone bodies, bilirubin, bile salt, nitrite, leukocyte esterase, potassium, urine appearance (clear, cloudy/opalescent, high yellow color, mild yellow color, smoky red, brownish, orange, red, black, milky), urine volume, GFR, dialysis type, serum creatinine, serum phosphate, age, and gender), layer-1 server computing record (timestamp, patient ID, type of data, server ID, delay (in msec.), and response time), and layer-2 server computing record (timestamp, patient ID, block ID, blockchain ID, uncle count, transaction count etc.). All of this data is pre-processed before applying the cognitive and machine learning approach to analyze and generate a response to kidney devices.

#### 3.1.4. Wearable Kidney Model with Industrial Kidney Internet of Things (IKIoT) and Other Healthcare 4.0 Processes

Figure 4 shows the JaamSim model for wearable kidney attached in a larger domain-based industrial Internet of Things network named as IKIoT having cloud, edge, cognitive, machine learning services for system efficiency suitable for larger distance (across the city, state or regions) hospital services.

### 3.2. Proposed Consensus Algorithm

In this section, a single and multi-player consensus algorithm is proposed for resource-constrained devices. Here, multiple consensus algorithms are proposed based on PoG. In PoG, either a single or multiple players observe specific activities of each other before considering the request of a node to add a new block in the existing blockchain.These proposed consensus algorithms are helpful in trust establishment as well. Here, past and present communication experiences help count the trust. This trust value increases with an increase in bits-matching. These algorithms are explained as follows.

#### 3.2.1. Single-Player Single-Bit (SPSB) PoG Consensus Algorithm

**Figure 5:**
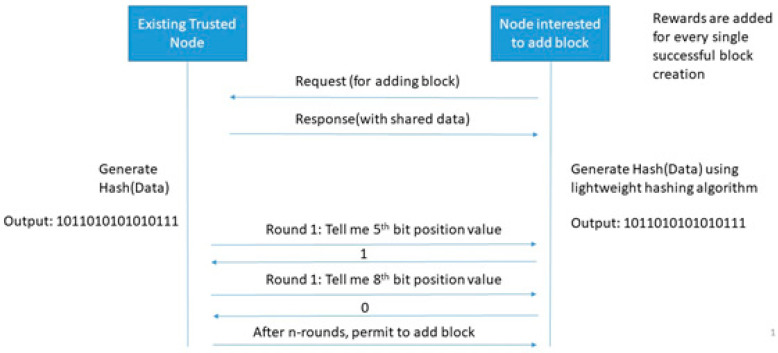
A proposed single-player single-bit PoG.

Figure 5 shows an example of the SPSB consensus algorithm. In this algorithm, a single bit of activity is observed. A simplified example if single bit activity is observing the position. For example, if there are two parties (existing trusted node and node interested to add new block) then SPSB PoG works in two phases: data exchange, and bit observation and verification phase. The data exchange phase share random data from an existing trusted node towards node interested to add a block. Thereafter, both sides take a computational challenge. For example, Figure 5 shows a ‘hash’ computational challenge. This challenge may vary from node to node but a common challenge should be selected at both sides. A publicly hidden challenge but known to two parties sound good for a healthy transaction system. Now, a set of activities are observed over a while. An existing trusted node can ask for a bit position at any time over this period and a node interested in adding a new block is strongly bound to reply to the bit position. After n-rounds of bit position verification, the node is allowed to add a block. In this complete process, node interested to add new blocks may contact multiple existing trusted nodes but get permission from the single existing trusted node. 

The probability of *i*^th^ node interested in adding block having successful bit matching with *N* individual existing trusted nodes out of *M*, where 1≤ i ≤ M−N+1, is computed using Equation (6):

(6) $$P\left(N_{i}\right)=\frac{1}{2^{N}}$$

Now, the probability of a single bit matching randomly from a single existing trusted node out of *M* is computed using Equation (7):

(7) $$P\left(N_{i}\right)=1-{\left(\frac{1}{2}\right)}^{M}$$

Further, the probability of success in a single bit matching after *K*-trials is computed using Equation (8):

(8) $$P\left(N_{i}\right)=HC\times (1-{\left(\frac{1}{2}{)}^{M}\right)}^{K}$$

where *HC* is the hit count i.e., the number of times *N* nodes are found active and the bit is matched. In this experimentation, *K*-trialsare made for reaching the desired level of successful hit counts.

**Figure 6:**
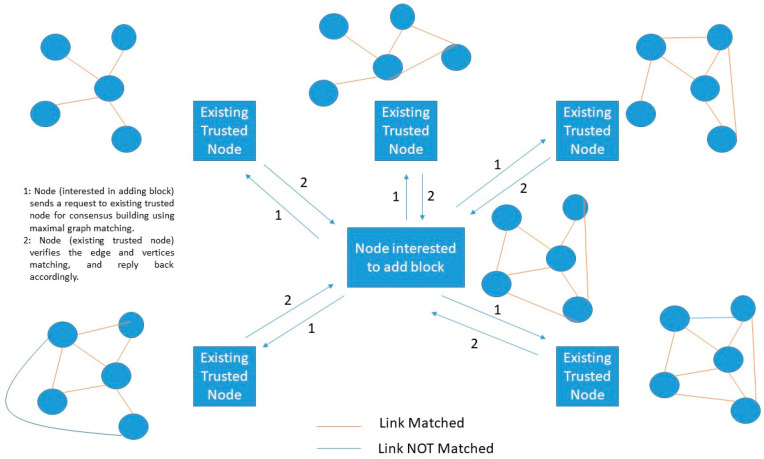
Proposed single player maximal graph (SPMG) PoG Consensus.

In this experimentation, the computational challenge may vary. For example, single-bit matching could be replaced with a maximal matching graph. Maximal graph matching computational challenge would allow all trusted nodes to select different graphs and omits the necessity of the data exchange phase. Figure 6 shows an example of a single-player maximal graph (SPMG) PoG consensus algorithm. In this algorithm, node interested in adding a new block selects a set of nearby existing trusted nodes having similar graph patterns (possibly the same number of vertices and edges). Further, there is one phase only in executing this algorithm: maximal edge matching. In maximal edge matching, node interested in adding block sends a request of edge matching to existing trusted nodes. If both parties found a maximal graph matching then-new block can be added. Now, the type of graph varies in this procedure. 

For example, the probability of success in the complete graph can be computed using Equation (9):

(9) $$P\left(N_{i}\right)=\frac{\left(2m\right)!}{2^{m}\times m!}$$

where *m* = 2*n* i.e., twice the number of vertices in the graph.

Although complexity varies with the computational challenge a new challenge procedure adds unknown computational efforts to a consensus algorithm that is healthy for consensus building.

#### 3.2.2. Multi-Player Single-Bit (MPSB) PoG Consensus Algorithm

**Figure 7:**
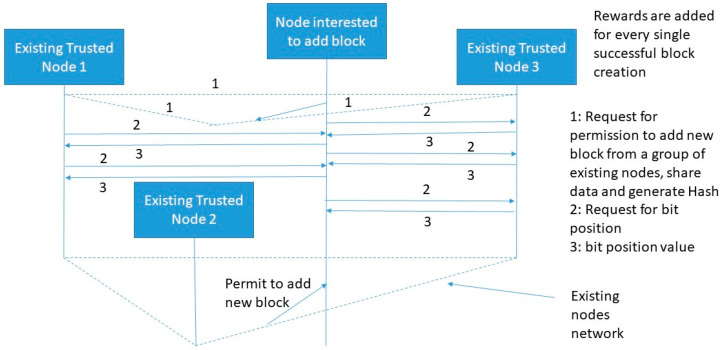
Proposed multi-player single-bit (MPSB) PoG consensus algorithm.

Figure 7 shows an example of the MPSB PoG consensus algorithm. In this case, multiple existing trusted players need to be satisfied before allowing any node to add a new block. 

A threshold number should be set for all nodes interested to add new blocks. This threshold factor is a performance factor that indirectly indicates the response time of creating a new block in the existing blockchain network. To speed-up the new block creation process, a single bit challenge for multiple players is the best criterion because it reduces real-time computational efforts and increases the performance as well. Now, suppose there are n-players interested to permit a node in adding a block and $R_{1}$, $R_{2}$, $R_{3}$, … $R_{n}$ represents the number of randomly matched bits for n-players at a particular time. Now the probability of allowing a node to add block from an individual player can be computed as: $p_{1}=2^{R_{1}/M},p_{2}=2^{R_{2}/M},p_{3}=2^{R_{3}/M}\ldots .\;p_{n}=2^{R_{n}/M}$. Here, *M* is the maximum number of bits available for matching. The success probability of node interested to add block is computed using Equation (9).

#### 3.2.3. Multi-Player Multi-Bits (MPMB) PoG Consensus Algorithm

**Figure 8:**
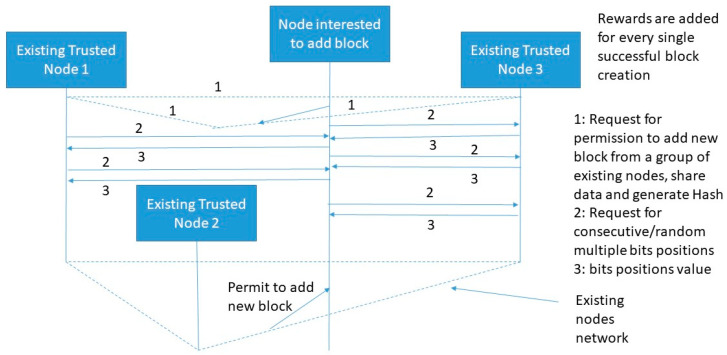
Proposed multi-player multi-bits (MPMB) PoG consensus algorithm.

The MPMB PoG consensus algorithm ensures consensus of multiple players after multi-bits matching between node interested to add a block and existing trusted node as shown in Figure 8. In multi-bits matching, bits can be selected randomly or consecutively. Both of these mechanisms increase computational complexity as compared to SPSB or MPSB. Random selection of multiple bits provides random bit matching every time which in-results to random challenge. Now, given a space of *N_1_* and *N_2_* possible number of bits picked from two sides (existing trusted node and node interested to add block) out of *M_1_* and *M_2_*. The probability of randomly generating multiple same bits is *N_1_/N_2_.* Where, *N_1_* ≤ *N_2_*. If *N*_1_ and *N*_2_ are of fixed size then the probability of randomly selecting *k*-multiple bits that are all matching is defined by Equation (10):

(10) $$\frac{N_{1}}{N_{2}}\times \frac{N_{1}-\left(k-1\right)}{N_{2}-\left(k-1\right)}\times \frac{N_{1}-\left(k-2\right)}{N_{2}-\left(k-2\right)}\times \ldots \times \frac{N_{1}-1}{N_{2}-1}$$

Variation in Equation (5) is exponential. As consent from multiple existing trusted nodes increases the probability of adding a block also increases. A highly randomized bits selection and matching generated unique challenge which in-turn increases the security level as well.

### 3.3. Proposed Simulation-Optimization in Improving Network Performances

In this section linear directional search approach-based simulation-optimization algorithms are proposed for identifying the optimum QoS based path for data transmission in the proposed Industry 4.0 processes-based approach. Algorithm 1 is linearly dependent on the previous set of QoS directions. There could be scenarios where QoS directions are linearly independent. For example, delay in transmission of a single packet between two ends it independent of delay in transmission from its predecessors. In such a linearly independent case, no linear search is required for identifying the optimum results. Further, both quadratic and non-quadratic set of problems can be solved with a maximum of *n*-iterations. Here, solutions of non-quadratic problems are different from quadratic problems because directions for further path selection are based on gradient or Hessian. Both gradient and Hessian simplifies the optimization process considerably for non-quadratic problems. Algorithm 2 is proposed for both quadratic and non-quadratic problem set suitable to be solved through gradient or Hessian methods. As compared to Algorithm 1, Algorithm 2 is time-consuming because of derivative calculations and it may not be suitable for those whose derivatives are impossible to deduce or compute. In specialized derivative cases, other mathematical computations (inversion, dot product, multiplication etc.) may be required for removing the errors or inaccuracies. Overall, it would be a significantly time-consuming process and should be avoided if critical services such as overloaded servers, critical patient data, fast accessibility etc. are involved.
**Algorithm 1:** Proposed QoS-sequential Coordinate-descent Data Transmission Path Search Algorithm  **Goal:** To find QoS based data transmission path through an intermediate device’s local properties with sequential search directions and strict mathematical incidental relation between successive search directions.   **Premises:** Let f(x) is the objective function with *n*-QoS parameters vector $x={\left[x_{1},x_{2},\;\ldots ,\;x_{n}\right]}^{T}$, search direction vector $d_{l}={\left[0\;0\ldots 0\;d_{l}\;0\ldots 0\right]}^{T}$, α is the coefficient of search direction vector and it varies with the property of parameter (under consideration), $x^{lo}$ is the local-optimum parameter input with $f\left(x^{lo}\right)$ local-final objective function with acceptable output, $x^{go}$ is the global-optimum parameter input with $f\left(x^{go}\right)$ global-final objective function with acceptable output, $E^{n}$ is the Euclidean space for all parameters, and $\beta_{QoS}^{min}$ and $\beta_{QoS}^{max}$ are the minimum and maximum acceptable tolerance level for QoS parameter. Further, $T_{simulation}$ is the total simulation time for finding the global optimum solution.(1)The objective function is decided initially with:$$\underset{x}{\min}F=f\left(x\right),\;\mathrm{Here},\;x\in E^{n}\;\mathrm{Or}\;\underset{x}{\max}F=f\left(x\right),\;\mathrm{Here},\;x\in E^{n}$$(2)Initialize the QoS parameter tolerance levels i.e., $\beta_{QoS}^{min}$ and $\beta_{QoS}^{max}$.(3)**While**$T_{simulation}$ expires **do**(4)**For***k* in [1,2,3,…,n]:  Set $d_{k}={\left[0\;0\ldots 0\;d_{k}\;0\ldots 0\right]}^{T}$  **If** $f\left(x_{k}+\alpha d_{k}\right)$ is Minimum **then**   $\alpha_{k}=\alpha$   Set $x_{k+1}$ = $x_{k}$ + $\alpha_{k}d_{k}$   Compute $f_{k+1}=f\left(x_{k+1}\right)$   **If** $\beta_{QoS}^{min}$ ≤ ||$\alpha_{k}d_{k}$|| ≤ $\beta_{QoS}^{max}$ **then**    $x^{lo}=x_{k+1}$    $f(x^{lo})=f_{k+1}$    Exit   **End If**  **End If** **End For** **If** *k* == *n* **then**  **Set** $x_{1}$ = $x_{k+1}$  *k* = *k+1*  **Goto Step 5** **End For****End While**(5)$x^{go}=x^{lo}$(6)$f\left(x^{go}\right)=f(x^{lo})$(7)$f_{final=f\left(x^{go}\right)}$

**Algorithm 2:** Proposed QoS-sequential Coordinate-descent Data Transmission Path Search Algorithm with Gradient Information.**Goal:** To find QoS based data transmission path through an intermediate device’s local properties without sequential search directions and strict mathematical incidental relation with gradient information. **Premises:** In addition to premises defined in algorithm 1, g(x) is the gradient and H(x) is the Hessian defined for objective function f(x). Here, ∇ is the derivative of objective function w.r.t. all *n*-parameters and $\frac{\partial f}{\partial x_{k}}$ is the derivative of f w.r.t. objective function parameter $x_{k}$.(1)Initialize ∇=[ ](2)The objective function is decided initially with:$$\underset{x}{\min}F=f\left(x\right),\;\mathrm{Here},\;x\in E^{n}\;\mathrm{Or}\;\underset{x}{\max}F=f\left(x\right),\;\mathrm{Here},\;x\in E^{n}$$(3)Initialize the QoS parameter tolerance levels i.e., $\beta_{QoS}^{min}$ and $\beta_{QoS}^{max}$.(4)**If**f(x) is first order derivative **then** **For** *k* in [1,2,3,…,n]:   Compute $\nabla^{k}=\frac{\partial f}{\partial x_{k}}$   Append ∇ with $\nabla^{k}$ **End For** **Compute** $\nabla =\nabla^{T}$ g(x)=∇f(x)**End If****If** f(x) is second order derivative **then** **For** *j* in [1,2,3,…,n]:   **For** *k* in [1,2,3,…,n]:    Compute $\nabla^{j,k}=\frac{\partial f}{\partial x_{j}\partial x_{k}}$    Append ∇ with $\nabla^{j,k}$    $H\left(x\right)=\nabla \left[\nabla^{T}f\left(x\right)\right]$   **End For** **End For****End If**(5)**For***k* in [1,2,3,…,n]: Compute $\alpha_{k}=\frac{{\left[g\left(x_{k}\right)\right]}^{T}g\left(x_{k}\right)}{{\left[d_{k}\right]}^{T}H_{k}\left(x\right)d_{k}}$ Set $x_{k+1}$ = $x_{k}$ + $\alpha_{k}d_{k}$ Compute $f_{k+1}=f\left(x_{k+1}\right)$**End For**(6)**If**$\beta_{QoS}^{min}$ ≤ ||$\alpha_{k}d_{k}$|| ≤ $\beta_{QoS}^{max}$ **then**
 $x^{lo}=x_{k+1}$ $f(x^{lo})=f_{k+1}$ Exit**End If**(7)Set k = k+1(8)Compute $g\left(x_{k+1}\right)=\nabla f\left(x_{k+1}\right)$(9)Compute $\alpha_{k}=\frac{{\left[g\left(x_{k+1}\right)\right]}^{T}g\left(x_{k+1}\right)}{{\left[g\left(x_{k}\right)\right]}^{T}g\left(x_{k}\right)}$(10)Compute $d_{k+1}=-g\left(x_{k+1}\right)+\alpha_{k}d_{k}$(11)Compute $\alpha_{k}=\frac{{\left[g\left(x_{k}\right)\right]}^{T}g\left(x_{k}\right)}{{\left[d_{k}\right]}^{T}H_{k}\left(x\right)d_{k}}$(12)Set $x_{k+1}$ = $x_{k}$ + $\alpha_{k}d_{k}$(13)Compute $f_{k+1}=f\left(x_{k+1}\right)$(14)**If**$\beta_{QoS}^{min}$ ≤ ||$\alpha_{k}d_{k}$|| ≤ $\beta_{QoS}^{max}$ **then** $x^{lo}=x_{k+1}$ $f(x^{lo})=f_{k+1}$ Exit **End If**(15)Go to step 7

## 4. Simulation and Results Analysis

In this section, blockchain is constructed and various proposed PoG consensus algorithms are integrated for performance and security analysis. These analyses are performed as follows:

(1) Kidney Operation Simulation and Analysis

**Figure 9:**
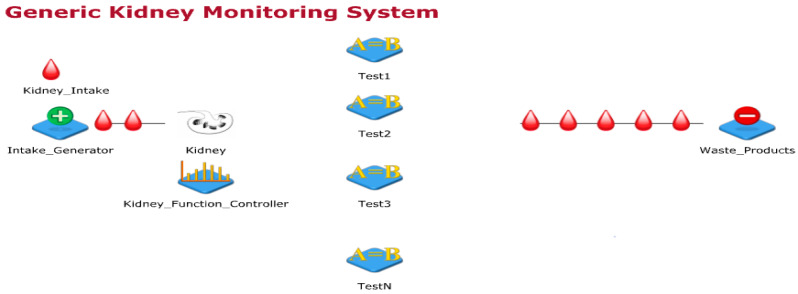
Generic kidney monitoring system in execution.

**Figure 10:**
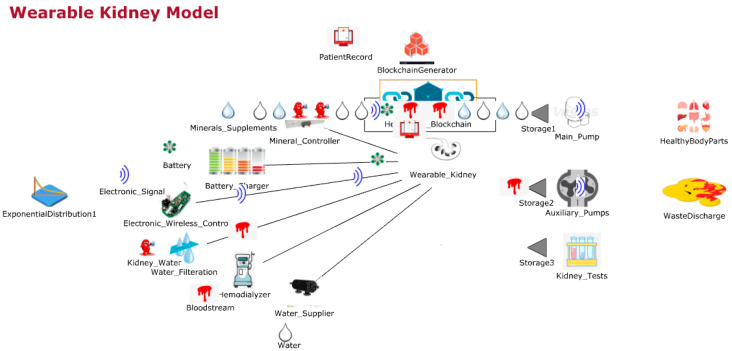
Lightweight and wearable kidney monitoring system in execution.

**Figure 11:**
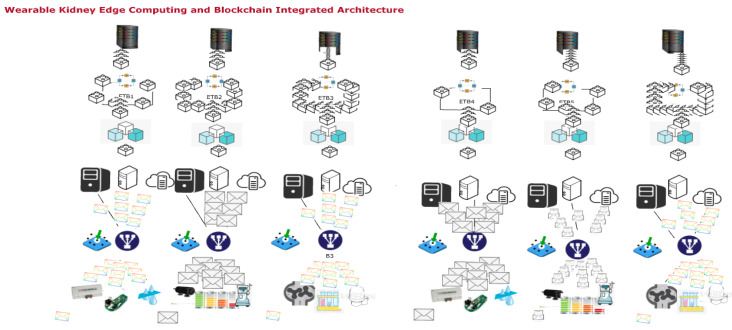
Proposed wearable kidney edge computing with IoT and blockchain technology in execution.

Figure 9 and Figure 10 show the execution of a generic kidney model and wearable kidney model. The kidney operations are simulated with different network scenarios starting from edge, fog and cloud network services with blockchain and Healthcare 4.0 processes as shown in Figure 11. To measure the performance, multiple case-studies with variations in patient type, disease identification, and treatment are taken in this work. These case studies are briefly discussed as follows:

*Case Study 1*: In this case, random 60 or more years-old patients with high blood pressure, frequent hypertensive complaints, eye infections, mild or complete mouth saliva absence, and frequent back pain are generated. Here, random variations of blood pressure (120 to 160)/(75 to 100) mm Hg, pulse range of (80 to 100)/min are generated. Among tests, blood sugar (both fasting and random) measurements range from (200 to 320) mg/dL, urine benedict (blue, green, orange and brick-red), urine albumin-to-creatinine ratio (2 to 30 mg/g), serum creatinine levels 0 to 6 mg/dL, BUN 7 to 130 mg/dL are simulated. Here, it is assumed that patients’ other tests including serum immunoglobulins, bacteria culture, serum electrophoresis etc. are normal. In treatment, a patient’s age, gender, and degree of renal damage are important to observe at the time of starting any treatment. There is need to go with other tests like protein loss, edema, hypercoagulable, mineral deficiencies and medication suitable for protection from side-infections. All of the medical test values are probabilistically generated randomly with JaamSim probabilistic distribution components. There is no constraint put over generating the number of patients or hospital referred.

• Kidney operation simulation with edge computing, IoT and blockchain network

**Figure 12:**
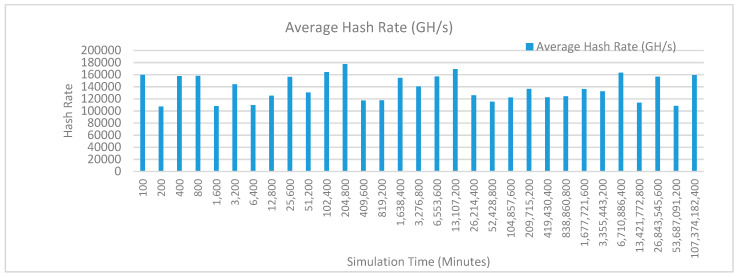
Average hash rate analysis (with IoT and edge).

Figure 12 shows the average hash rate variations for the proposed approach with edge, IoT, and blockchain (with multi-player single-bit PoG) technologies. The hash rate varies between 0.1 million GH/s to 0.18 million GH/s. The trends of hash rate are variable over simulation time because it depends upon incoming data and transactions. In simulation data and transactions are uniformly and continuously planned thus its hash rate is not constant.

• Kidney operation simulation with edge computing, fog computing, IoT and blockchain network

**Figure 13:**
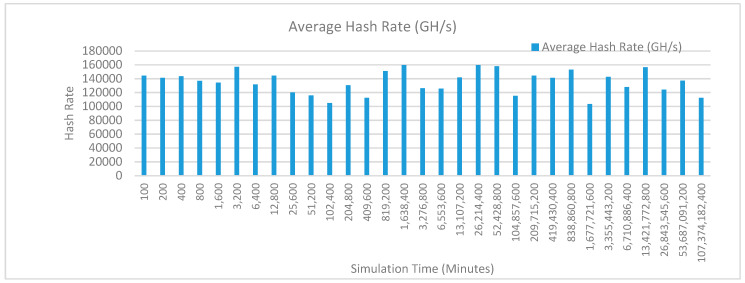
Average hash rate analysis (with IoT, edge, fog).

Figure 13 shows the average hash rate variations for the proposed approach with edge, fog, IoT, and blockchain (with multi-player single-bit PoG) technologies. The hash rate varies between 0.1 million GH/s to 0.16 million GH/s. The maximum range of hash rate, in this case, is lower than the previous case (kidney operation simulation with edge computing, IoT and blockchain network) because fog computing added additional time overhead which in-turn increases the time taken to commit a transaction and thus, reduces the hash rate as well. The trends of hash rate are variable over simulation time because it depends upon incoming data and transactions. In simulation data and transactions are uniformly and continuously planned thus its hash rate is not constant.

• Kidney operation simulation with edge computing, fog computing, cloud computingIoT and blockchain network

**Figure 14:**
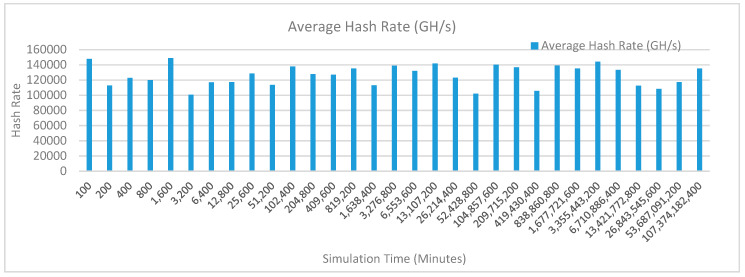
Average hash rate analysis (with IoT, edge, fog, and cloud).

Figure 14 shows the average hash rate variations for the proposed approach with edge, fog, IoT, and blockchain (with multi-player single-bit PoG) technologies. The hash rate varies between 0.1 million GH/s to 0.15 million GH/s. 

The maximum range of hash rate, in this case, is lower than the previous two cases (kidney operation simulation with edge computing, IoT and blockchain network, and kidney operation simulation with edge computing, fog computing, IoT and blockchain network) because fog and cloud computing added additional time overhead which in-turn increases the time taken to commit a transaction and thus, reduces the hash rate as well. The trends of hash rate are variable over simulation time because it depends upon incoming data and transactions. In simulation data and transactions are uniformly and continuously planned thus its hash rate is not constant.

*Case Study 2*: In this case, random 40 to 60 years old male patients with hemoglobin levels range from 11.5 to 18.5 g per deciliter, platelet range from 150,000 to 450,000 platelets per microliter of blood with a total count greater than 8000/cu. mm, and ESR 1 to 20 mm/h is considered. All other remaining parameters are considered to be the same as case study 1. In treatment, other laboratory tests including BUN, serum creatinine, hemoglobin, electrolytes, serological tests, RBC and WBC counts, urine electrolytes etc. are necessary to recommend a patient to go for a particular type of kidney treatment process. In the simulation, these values are probabilistically random and generated through the JaamSim model only.

• Kidney operation simulation with edge computing, IoT and blockchain network

**Figure 15:**
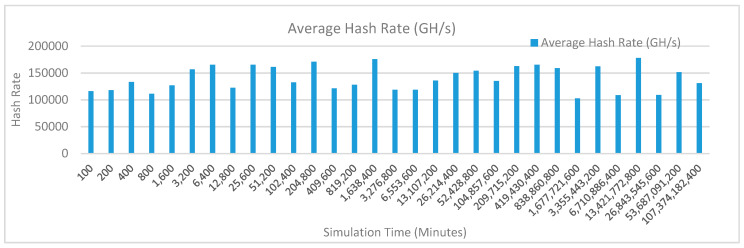
Average hash rate analysis (with IoT and edge).

Figure 15 shows the average hash rate variations for the proposed approach with edge, IoT, and blockchain (with multi-player single-bit PoG) technologies. The hash rate varies between 0.1 million GH/s to 0.18 million GH/s. The trends of hash rate are variable over simulation time because it depends upon incoming data and transactions. In simulation data and transactions are uniformly and continuously planned thus its hash rate is not constant.

• Kidney operation simulation with edge computing, fog computing, IoT and blockchain network

**Figure 16:**
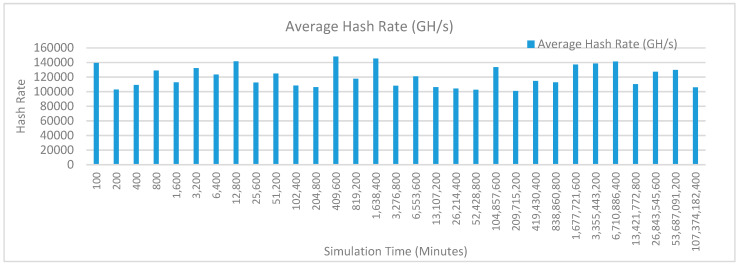
Average hash rate analysis (with IoT, edge, fog).

Figure 16 shows the average hash rate variations for the proposed approach with edge, fog, IoT, and blockchain (with multi-player single-bit PoG) technologies. The hash rate varies between 0.1 million GH/s to 0.16 million GH/s. The maximum range of hash rate, in this case, is lower than the previous case (kidney operation simulation with edge computing, IoT and blockchain network) because fog computing added additional time overhead which in-turn increases the time taken to commit a transaction and thus, reduces the hash rate as well. The trends of hash rate are variable over simulation time because it depends upon incoming data and transactions. In simulation data and transactions are uniformly and continuously planned thus its hash rate is not constant.

• Kidney operation simulation with edge computing, fog computing, cloud computingIoT and blockchain network

**Figure 17:**
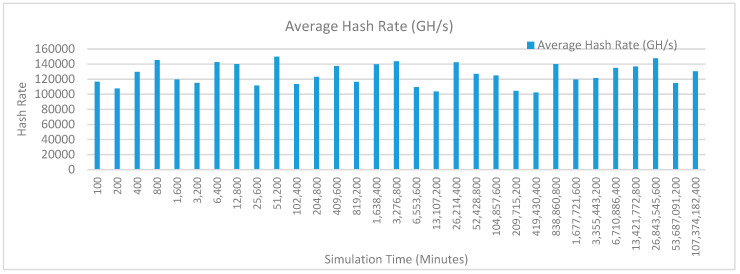
Average hash rate analysis (with IoT, edge, fog, and cloud).

Figure 17 shows the average hash rate variations for the proposed approach with edge, fog, IoT, and blockchain (with multi-player single-bit PoG) technologies. The hash rate varies between 0.1 million GH/s to 0.15 million GH/s. The maximum range of hash rate, in this case, is lower than the previous two cases (kidney operation simulation with edge computing, IoT and blockchain network, and kidney operation simulation with edge computing, fog computing, IoT and blockchain network) because fog and cloud computing added additional time overhead which in-turn increases the time taken to commit a transaction and thus, reduces the hash rate as well. The trends of hash rate are variable over simulation time because it depends upon incoming data and transactions. In simulation data and transactions are uniformly and continuously planned thus its hash rate is not constant.

*Case Study 3:* In this case, random 40 to 60 years old male patients with hemoglobin levels range from 11.5 to 18.5 g per deciliter, platelet range from 150,000 to 450,000 platelets per microliter of blood with a total count greater than 8000/cu. mm, and ESR 1 to 20 mm/h is considered. All other remaining parameters are considered to be the same as case study 1. These values are probabilistically random and generated through the JaamSim model only.

• Kidney operation simulation with edge computing, IoT and blockchain network

**Figure 18:**
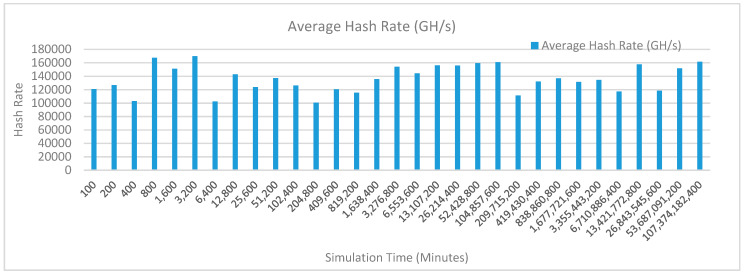
Average hash rate analysis (with IoT and edge).

Figure 18 shows the average hash rate variations for the proposed approach with edge, IoT, and blockchain (with multi-player single-bit PoG) technologies. The hash rate varies between 0.1 million GH/s to 0.17 million GH/s. The trends of hash rate are variable over simulation time because it depends upon incoming data and transactions. In simulation data and transactions are uniformly and continuously planned thus its hash rate is not constant.

• Kidney operation simulation with edge computing, fog computing, IoT and blockchain network

**Figure 19:**
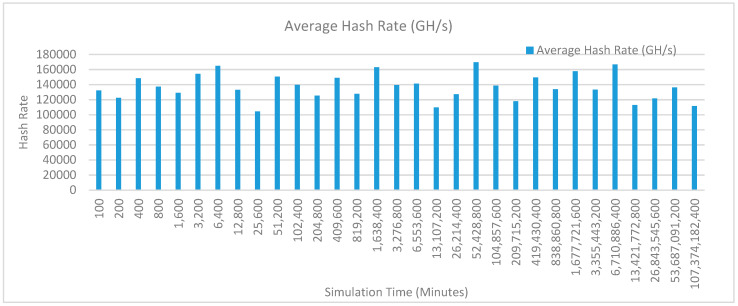
Average hash rate analysis (with IoT, edge, fog).

Figure 19 shows the average hash rate variations for the proposed approach with edge, fog, IoT, and blockchain (with multi-player single-bit PoG) technologies. The hash rate varies between 0.1 million GH/s to 0.17 million GH/s. The minimum and maximum range of hash rate, in this case, is same as of previous case (kidney operation simulation with edge computing, IoT and blockchain network) because the number of patients, in this case, is very large and resources are continuously engaged to have a high hash rate and delays are minimal. The trends of hash rate are variable over simulation time because it depends upon incoming data and transactions. In simulation data and transactions are uniformly and continuously planned thus its hash rate is not constant.

• Kidney operation simulation with edge computing, fog computing, cloud computingIoT and blockchain network

**Figure 20:**
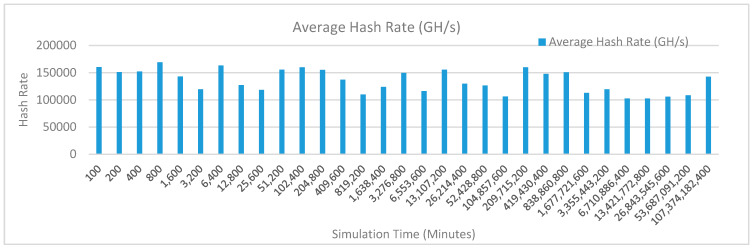
Average hash rate analysis (with IoT, edge, fog, and cloud).

Figure 20 shows the average hash rate variations for the proposed approach with edge, fog, IoT, and blockchain (with multi-player single-bit PoG) technologies. The hash rate varies between 0.1 million GH/s to 0.17 million GH/s. The minimum and maximum range of hash rate, in this case, is same as of the previous two cases (Kidney operation simulation with edge computing, IoT and blockchain network, and kidney operation simulation with edge computing, fog computing, IoT and blockchain network) because the number of patients, in this case, are very large and resources were engaged with transactions only that int turns reduces the delay and increases the hash rate. The trends of hash rate are variable over simulation time because it depends upon incoming data and transactions. In simulation data and transactions are uniformly and continuously planned thus its hash rate is not constant.

*Case Study 4*: A Caucasian 25 years old male presented with complaints of bilateral flank pain, hypertension, and intermittent hematuria. Family history is suggestive of some ongoing kidney issues in the family which is under-diagnosed as of early death in the family in the late 30 s. On examination, if he is noted to have bilateral irregular mass in the unilateral region. Bloods test performed including complete blood counts, urea, electrolytes, BUN, CRP, LFTs, etc. Renal USS reveals bilateral multiple cysts in the kidneys. Further evaluation including renal CT and intravenous pyelogram were performed. Treatment includes antibiotics treatment for UTIs, hypertensive, low sodium diet and regular follow up particularly brain CT to manage long term complications.

• Kidney operation simulation with edge computing, IoT and blockchain network

**Figure 21:**
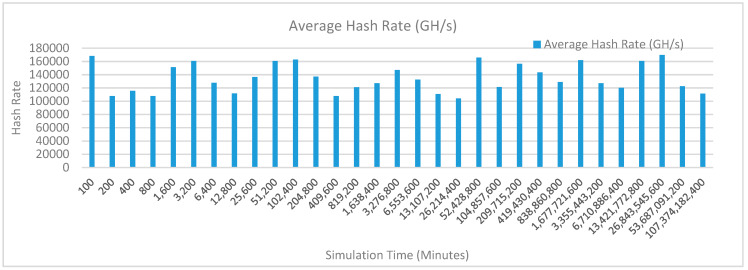
Average hash rate analysis (with IoT and edge).

Figure 21 shows the average hash rate variations for the proposed approach with edge, IoT, and blockchain (with multi-player single-bit PoG) technologies. The hash rate varies between 0.1 million GH/s to 0.17 million GH/s. The trends of hash rate are variable over simulation time because it depends upon incoming data and transactions. In simulation data and transactions are uniformly and continuously planned thus its hash rate is not constant.

• Kidney operation simulation with edge computing, fog computing, IoT and blockchain network

**Figure 22:**
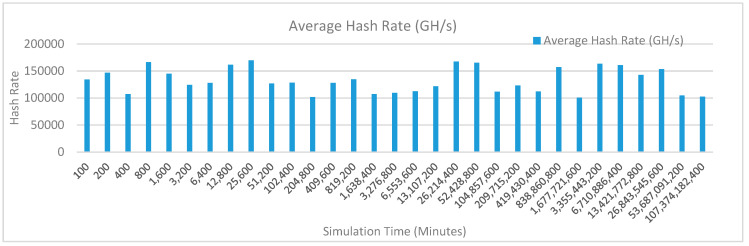
Average hash rate analysis (with IoT, edge, fog).

Figure 22 shows the average hash rate variations for the proposed approach with edge, fog, IoT, and blockchain (with multi-player single-bit PoG) technologies. The hash rate varies between 0.1 million GH/s to 0.17 million GH/s. The minimum and maximum range of hash rate, in this case, is same as of previous case (kidney operation simulation with edge computing, IoT and blockchain network) because the number of patients, in this case, is very large and resources are continuously engaged to have a high hash rate and delays are minimal. The trends of hash rate are variable over simulation time because it depends upon incoming data and transactions. In simulation data and transactions are uniformly and continuously planned thus its hash rate is not constant.

• Kidney operation simulation with edge computing, fog computing, cloud computing IoT and blockchain network

**Figure 23:**
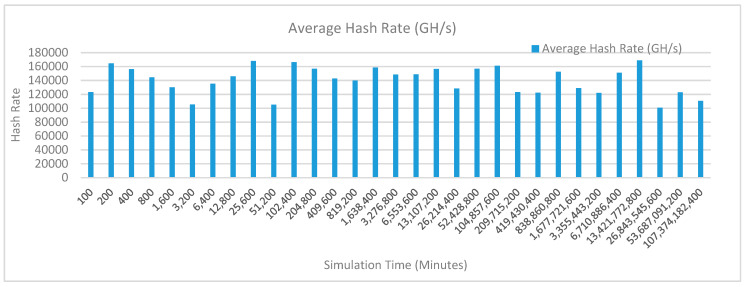
Average hash rate analysis (with IoT, edge, fog, and cloud).

Figure 23 shows the average hash rate variations for the proposed approach with edge, fog, IoT, and blockchain (with multi-player single-bit PoG) technologies. The hash rate varies between 0.1 million GH/s to 0.17 million GH/s. The minimum and maximum range of hash rate, in this case, is same as of the previous two cases (kidney operation simulation with edge computing, IoT and blockchain network, and kidney operation simulation with edge computing, fog computing, IoT and blockchain network) because the number of patients, in this case, are very large and resources were engaged with transactions only that int turns reduces the delay and increases the hash rate. The trends of hash rate are variable over simulation time because it depends upon incoming data and transactions. In simulation data and transactions are uniformly and continuously planned thus its hash rate is not constant.

*Case Study 5*: A young 21 year-old female presented with sudden onset of fever, crampy abdominal pain and purpuric rash starting on the legs. After hospital admission, she has initial investigations including full blood count (FBC), U &E, BUN, clotting screen, LFTs, urine culture and clood cultures. Details of personal history revealed recent respiratory infection. This patient is observed with purpura and it is a Henoch-Schonlein Purpura (HSP) hallmark and there I no chance of it due to low platelet count or any inflammation. This patient is put under observations and abnormalities include arthritis, abdominal disturb, kidney inflammation etc. are frequently observed. These signs are found to be observed majorly in children very frequently. In children, a disease with similar signs is resolved much earlier as compared to adults. Now, the patient should be cross-examined for a renal disease before prescribing for any nonsteroidal anti-inflammatory drug. A 100 mg/day dapsone dose can be prescribed for HSP positive case considering that it can affect the abnormal immune system response. Steroids are not preferred choice. It is recommended to discuss with the patient that in recurrent spells of purpura patients the chances of resolving it of its own are much higher.

• Kidney operation simulation with edge computing, IoT and blockchain network

**Figure 24:**
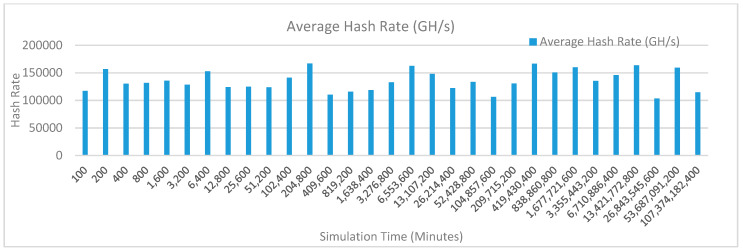
Average hash rate analysis (with IoT and edge).

Figure 24 shows the average hash rate variations for the proposed approach with edge, IoT, and blockchain (with multi-player single-bit PoG) technologies. The hash rate varies between 0.1 million GH/s to 0.17 million GH/s. The trends of hash rate are variable over simulation time because it depends upon incoming data and transactions. In simulation data and transactions are uniformly and continuously planned thus its hash rate is not constant.

• Kidney operation simulation with edge computing, fog computing, IoT and blockchain network

**Figure 25:**
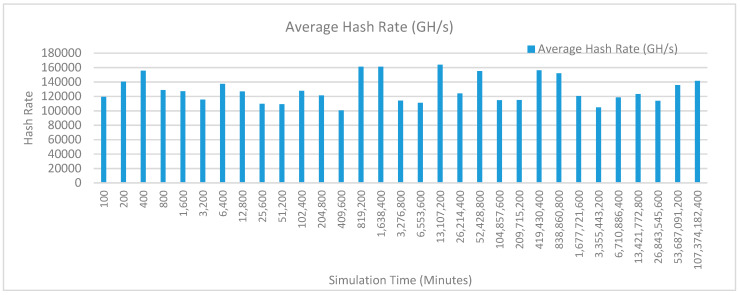
Average hash rate analysis (with IoT, edge, fog).

Figure 25 shows the average hash rate variations for the proposed approach with edge, fog, IoT, and blockchain (with multi-player single-bit PoG) technologies. The hash rate varies between 0.1 million GH/s to 0.17 million GH/s. The minimum and maximum range of hash rate, in this case, is same as of previous case (kidney operation simulation with edge computing, IoT and blockchain network) because the number of patients, in this case, is very large and resources are continuously engaged to have a high hash rate and delays are minimal. 

The trends of hash rate are variable over simulation time because it depends upon incoming data and transactions. In simulation data and transactions are uniformly and continuously planned thus its hash rate is not constant.

• Kidney operation simulation with edge computing, fog computing, cloud computing, IoT and blockchain network

**Figure 26:**
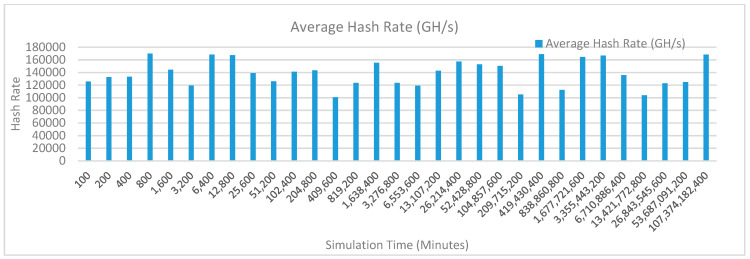
Average hash rate analysis (with IoT, edge, fog, and cloud).

Figure 26 shows the average hash rate variations for the proposed approach with edge, fog, IoT, and blockchain (with multi-player single-bit PoG) technologies. The hash rate varies between 0.1 million GH/s to 0.17 million GH/s. The minimum and maximum range of hash rate, in this case, is same as of the previous two cases (kidney operation simulation with edge computing, IoT and blockchain network, and kidney operation simulation with edge computing, fog computing, IoT and blockchain network) because the number of patients, in this case, are very large and resources were engaged with transactions only that int turns reduces the delay and increases the hash rate. The trends of hash rate are variable over simulation time because it depends upon incoming data and transactions. In simulation data and transactions are uniformly and continuously planned thus its hash rate is not constant.

• Learning points from kidney simulations and case-studies:

The following are the other learning points from kidney simulation and medical case studies.

**Figure 27:**
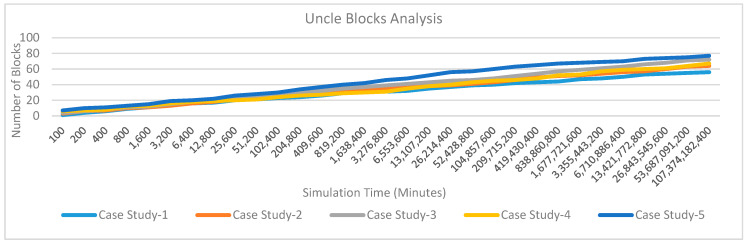
Comparative uncle block analysis.

*Uncle Block Analysis*: Uncle blocks are not part of the blockchain network family but they are potential blocks of the network. Figure 27 shows the number of uncle blocks variations over simulation time for five case studies. It is observed that the case study 4 and case study 5 are having higher uncle block variations as compared to case study 1 to case study 3. This is because case study1, case study 2 and case study 3 are uncommon and a large number of tests are required before considering the patient under some treatment. Each of these tests requires one or more transactions and these transactions delay the execution. Whereas, case study 4 and case study 5 predicts normal patients. These cases are large in number and there are multiple block competitors to complete the transactions which in-turn increases the number of uncle blocks. The creation of uncle blocks shows healthy competition that in-turns reflect blockchain healthiness. As a result, it is observed that the proposed blockchain approach and its integration with industry 4.0 processes show a good quality network and interoperability.

**Figure 28:**
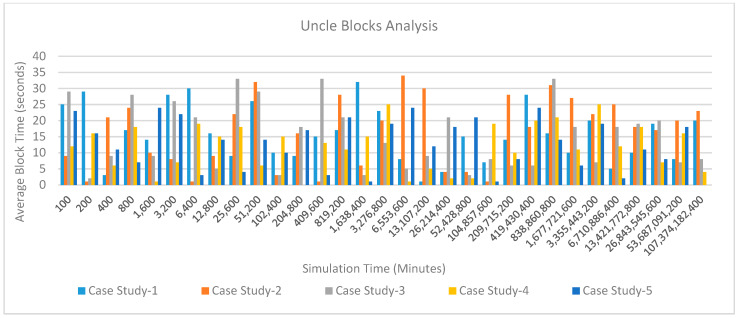
Average block size variations over simulation time for five case-studies.

*Block Size Variations*: Figure 28 shows the average block time variations over simulation time for five case studies. Results show that the average block time in case stud 4 and case stud 5 is comparatively lesser than case study 1, case study 2 or case study 3. This is because of the number of transactions required in the case of case study 4 and case study 5 is much lesser than other case studies. These transactions are lesser because the days required to undergo treatment, medication and doctor consultations are lesser as compared to other cases. In the case of other case studies (case study 1, case study 2 and case study 3), the block time variation is very large because the type of treatment varies from patient to patient and all of these cases are sensitive and specialized cases of kidney treatment. Thus, special care is required to treat them. In their treatment, a large amount of data and associated transactions are required which in-turn increases the block size.

(2) Security Analysis

In the security analysis, themajority attack is analyzed statistically. This analysis is explained as follows:

*Majority Attack*: In single or multiple existing trusted node scenarios, if attackers are present and these attacks have more control over existing trusted nodes or they have their own existing trusted nodes more than other nodes then a majority attack can happen by incorporating wrong transactions during mining. For example, SPSB PoA accepts the consensus of one player only before allowing any node to add a block. Now, if an attacker incorporates his node in the network and any node places a request to this node for adding a block then chances of an attacker in fulfilling his intentions are higher. As the number of players required for consensus-building increases, the chances of a majority attack decrease. As a result, if the number of blocks in blockchain and trust of participants in the blockchain network increases then it would be difficult to break ‘proof-of-game’ for an attacker and tamper the chain. In this work, a Chernoff bound equation is used to prove this concept. Here, if *P* is the population size of honest participants that are using proposed ‘proof-of-game’ for consensus building and q-dishonest participants are trying to break the game with tampering. Now, both (honest and dishonest) participants would try to maximize their chances of success ($C^{S})\;$ as:

(11) $$C_{M}^{S}=Max\left\{{\left\{Uniform\left(0,1\right)\right\}}^{P}\right\}$$

(12) $$C_{m}^{S}=Max\left\{{\left\{Uniform\left(0,1\right)\right\}}^{q}\right\}$$

Let’s assume that there are *n*-blocks created in the blockchain. Thus, the probability of blockchain construction ($P_{const}^{n})$ with high trust can be computed as:

(13) $$P_{const}^{n}=\overset{n}{\underset{i=1}{\sum }}C_{i}^{S}-C_{q}^{S}$$

On the other hand, Chernoff bound and independence gives the probability that the attacker is successfully able to tamper the blockchain and it is computed as follows:

(14) Ptamper(CNS≤0)≤Es>0min[e−sCNS]=∏t=1Ns>0minE[eCPS(t)]E[eCqS(t)]=(E[eCMS(t)]E[eCmS(t)])Ns>0min

Here, successful blockchain construction means *P* > q and *s* > 0. In Equation (14) any product is the inner dot product and its expectations are less than 1. Equation (14) also shows that the success probability of tampering a blockchain decreases if the number of blocks in blockchain increases or the trust of participants is continuously building upward.

*Other Attacks*: In our previous [43,44,45,46], other attack analysis such as man-in-the-middle, wormhole, black-hole, privacy leakage and synchronization failure-based attacks are explored against lightweight bit verification approaches. It is observed that the proposed approach is secure against all of these attacks because of fast bit-verification approach and randomization.

(3) Overall Blockchain Network Analysis

This sub-section shows overall blockchain network performance analysis using (i) priority of blocks, (ii) block round and confirmation time, and (iii) number of blocks mined. The explanations are as follows.

**Figure 29:**
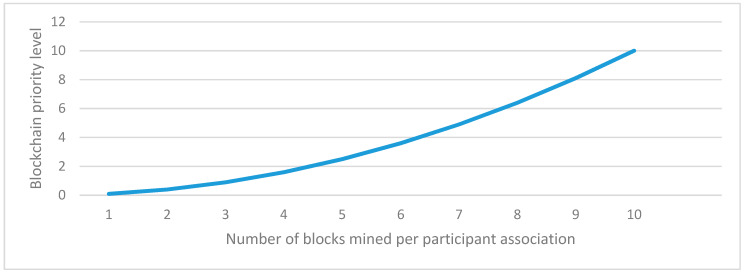
Comparative analysis of blockchain priority level and number of blocks mined per participant.

*BlockchainPriority Level*: As observed earlier, the increase in participant’s trust over blockchain makes the security level high. A similar trend is observed in the number of players playing the game and their association with an increase in the priority level of the blockchain network. If any participant is considered as an authentic user of the blockchain network then the chances of his/her playing the game in the network are also very high which in-turn increases the number of blocks mined. Figure 29 shows this presentation. Although this variation is slightly lesser than linear variation but it gradually increases towards a larger number.

**Figure 30:**
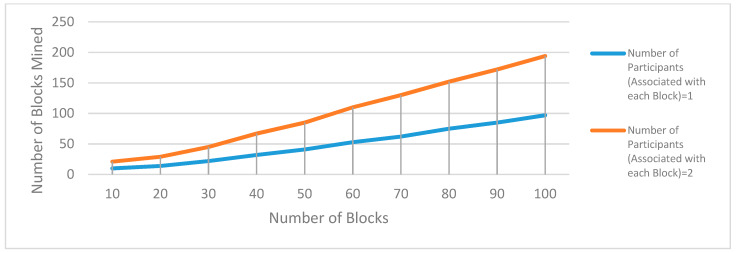
Analysis of the number of blocks mined as the number of participants associated with those blocks increases with time.

*Block’s round time and confirmation time*: It is observed that the block’s round time and confirmation timeis lesser than 10 min. This period is lesser because the resources available are varying with challenge generation and confirmation. The propagation time is also close to real propagation time of the Bitcoin network. Thus, the simulated is validated. Figure 30 shows an analysis of participants association with blocks. Results show that the number of blocks mined increases with an increase in participants association with blocks. Here, two scenarios are taken into consideration. In the first scenario, one-participant is associated per block and in the second scenario, two-participants are associated with blocks. The number of blocks mined increases at a much higher rate for the two-participants scenario as compared to the one-participant scenario.

**Figure 31:**
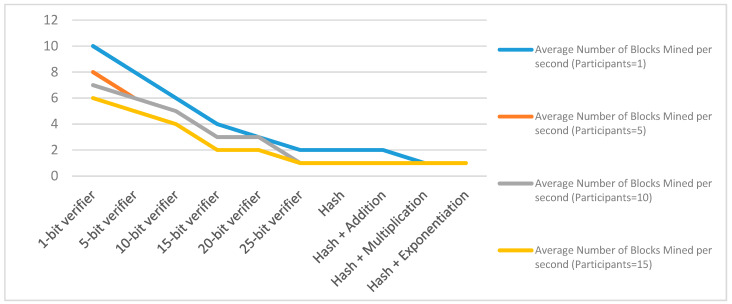
Comparative analysis of change in the number of blocks mined per seconds with a change in block challenges.

*The number of blocks mined per second*: An analysis over Intel Core i7-7200U CPU@2.5GHz, 8 GB RAM and the 64-bit operating system shows that the number of blocks mined decreases exponentially with an increase in bit-based or other increasing complexity based computational challenges as shown in Figure 31. Here, 1, 5, 10, 15, 20 and 25-bits verification challenges are taken into consideration for the bit-verification scenario. The other category of challenge generation scenario is based on a hash function.

(4) Proposed PoG Consensus Algorithms Performance Analysis with Kidney System

**Figure 32:**
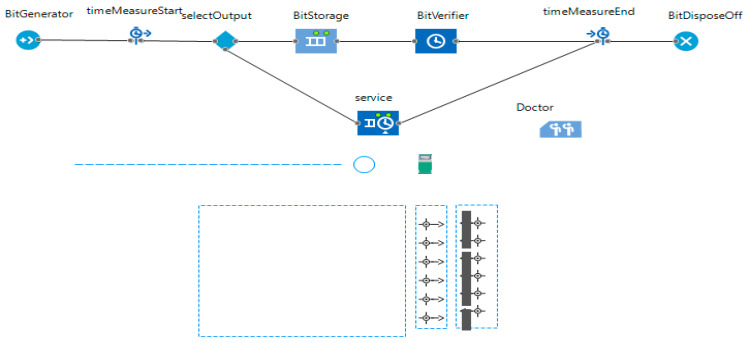
AnyLogicPoG Bit Verifier Circuit for Patient-Doctor Model.

Figure 32 shows the AnyLogic proposed PoG bit verifier circuit for automated Patient-Doctor wearable kidney interactions [47,48,49,50]. This circuit shows the integration of two different systems (i) the proposed PoG-based consensus model with variable bit-based challenge verification, and (ii) the healthcare system proposed for the wearable kidney system. The major components of the circuit are explained as follows.

- *BitGenerator*: This component generates fixed length but random bits. These bits are used at both sides (source and destination) for verification later.
- *timeMeasureStart*: This is the start time when the bits-matching system is observed for performance analysis.
- *selectOutput*: This component is to divide the generated bits equally and parallel with the doctor. This division is later used for verification between the wearable kidney and the doctor service to build consensus.
- *BitStorage*: Here, bits are stored in the patient’s wearable kidney electronic system. Total-bits length is fixed and it does not have large memory requirements. Thus, the processes are light in terms of computational and communicational costs.
- *BitVerifier*: These components pick bits from both the doctor component and the patient’s wearable kidney’s *BitStorage* component for comparison. In the simulation, a delay component is used for this work. It adds a variable delay corresponds to bit comparison time.
- *timeMeasureEnd*: This is the end time of system observations. Both timeMeasureStart and timeMeasureEndare used in various performance measurements such as circuit throughput, delay, jitter etc.
- *BitDisposeOff*: This simulation circuit is designed to continuously generate new bits. Thus, previously generated bits from both the wearable kidneys’ *BitStorage* and the doctor service are disposed of using this component.
- *Service*: This is the doctor’s service component. This component associates’ doctor with the circuit.
- *Doctor*: A doctor is considered to be a person handling patients medically. It provides its services through the *service* component.

**Figure 33:**
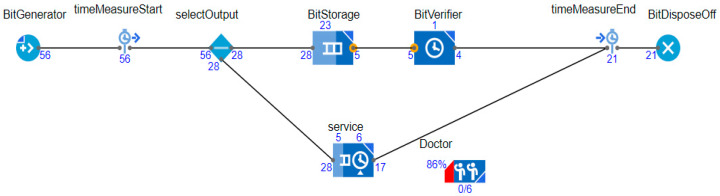
AnyLogicPoG Bit Verifier Circuit for Patient-Doctor Model in Execution.

There are other circuit components shown in Figure 32 as well such a dotted line (to follow the queue while treatment), small circle (a position where patient stand for hospitalization), large rectangle area (showing patient waiting area, and small rectangles with position symbols and tables (representing patient and doctors).These components are better explained with 2D or 3D model execution after results discussions. Figure 33 shows the AnyLogicPoG bit verifier circuit for patient-doctor interaction and treatment model in execution.

**Figure 34:**
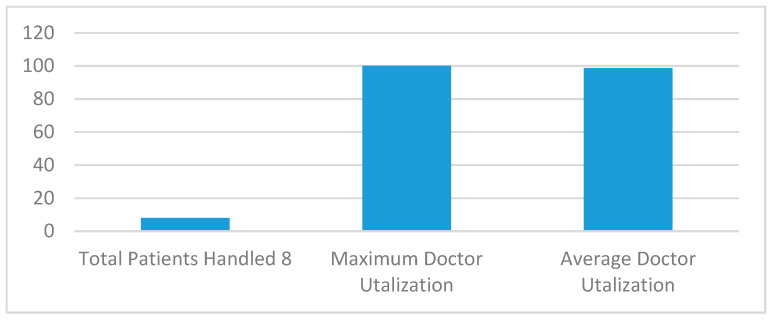
AnyLogic Graph for Patients and Doctor Statistics.

Figure 34 shows the statistical graphs computing total patient handled, maximum doctor utilization, and average doctor utilization. These statistics are dynamic i.e., variable with simulation time. Figure 34 shows one view after 3 s. At this time there are eight patients handled by 6 doctors with 100% doctor utilization (i.e., all are available and working) and average doctor utilization is 98.62%. Average doctor utilization is less than 100% because of the doctor’s other activities (excluding patient handling). All the timing parameters in the system are scaled down to 1/100th for fast execution.

**Figure 35:**
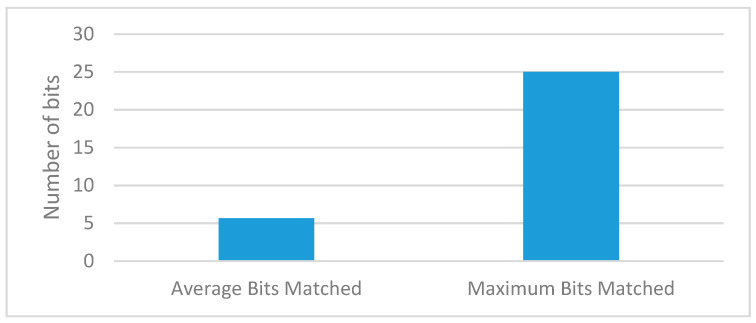
AnyLogic Statistics for Bits Verifications in PoG.

Figure 35 shows the average bits matched in the proposed PoG consensus model using AnyLogic simulation. Results show that the average bits matched are 5.66 and maximum bits matched are 25 after 3 s. This shows that the proposed PoG consensus model is having less time complexity. The average bits matched are not an exact number because some bits are in the process of matching.

**Figure 36:**
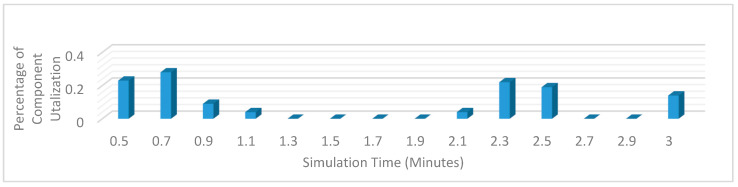
System Working Distribution Variations with simulation time.

Figure 36 shows the system working distribution variation with simulation time. System working distribution is defined as the variation presenting the percentage of component utilization over simulation time. Like Figure 34, Figure 35 and Figure 36 is also dynamic i.e., its values are continuous changes with simulation time. Figure 36 shows one screenshot during the initial 3 s. Results show that the maximum utilization of around 28.5% between 1 and 1.5 s.

**Figure 37:**
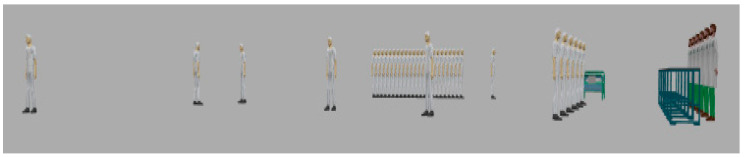
AnyLogic Patient-Doctor Simulation 3D Model in Execution (side viewpoint).

**Figure 38:**
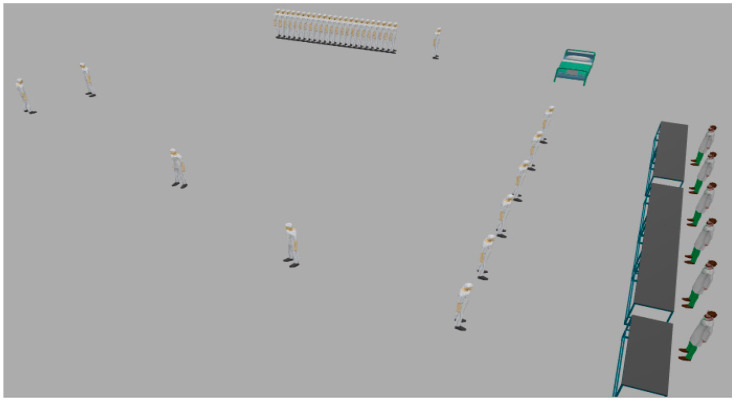
AnyLogic Patient-Doctor Simulation 3D Model in Execution (top viewpoint).

**Figure 39:**
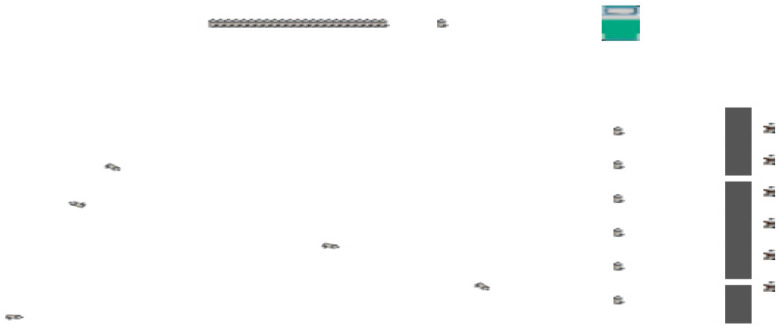
AnyLogic Patient-Doctor Simulation 2D Model in Execution (top viewpoint).

Figure 37 shows the AnyLogic patient-doctor simulation in 3D view from one side. This diagram shows that the patients are waiting in the waiting area before they queue-up for treatment from a doctor. Figure 38 shows the top-view of 3D model designed for patient-doctor interaction. It looks like the patient is physically interacting with doctors but this is a simulation only. In real-scenario, patients with wearable kidney interact with the doctor through proposed wearable kidney with IoT, edge computing and blockchain technology. Figure 39 is the patient-doctor simulation model in 2D.

(5) Proposed Healthcare 4.0 Processes-based Network’s Performance Analysis

**Figure 40:**
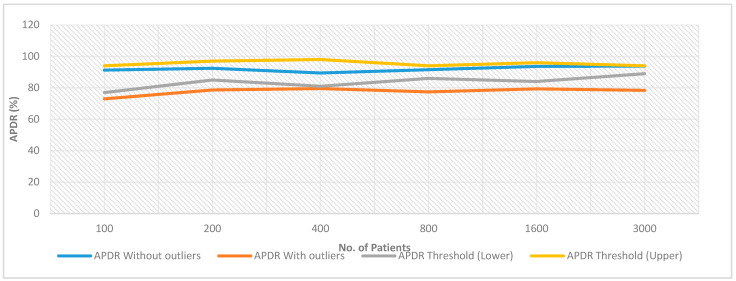
Average Packet Data Rate (ADPR) for Proposed IoT-Sensor based Network.

Figure 40 shows the Average Packet Delivery Rate (APDR) with an increase in patients in the proposed IoT-Sensor-based healthcare system. These patients are generic kidney patients and the performance of the network is analyzed for data storage and accessibility. Here, APDR is defined as the ratio of packets successfully received to the destination divided by a packet sent by a source for medical record storage or accessibility. Results show that the observed ADPR (without outliers) lies between upper and lower acceptable limits. These upper and lower acceptable limits are decided based on maximum and minimum data required (for storage and accessibility) for generic patients respectively. The observed APDR lies between 95% (approx.) to 97% (approx.). Thus, the proposed IoT-sensor-based framework is considered to be efficient in performance. ADPR (with outlier) lies below threshold limits. These outliers are considered to be present in the network for data breaches. These outlier activities increase the number of packets in the network and lower the performance of patient data storage or accessibility. Thus, overall network performance decreases. As the ADPR percentage decreases below the lower acceptable range, the network is considered under attack. Thus, the security level can be increased with an increase in the number of bits matching for consensus building.

**Figure 41:**
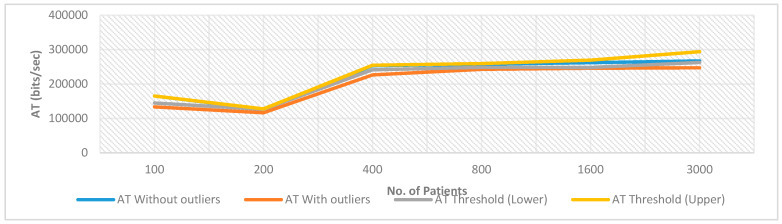
Average throughput (AT) for Proposed IoT-Sensor based network.

Figure 41 shows the average throughput (AT) variations with an increase in the number of patients. AT is defined as the average value of total payload (bits) successfully delivered to the destination node divided by simulation time in the network. Results show that the AT (without outlier) is lying between upper and lower acceptable limits. Further, AT (with outliers) is below the lower threshold limit. This indicates that attackers (with data breach) activities can lower the network performance and can be identified easily. The lower and upper AT values lie between 120 Kbps to 250 Kbps, approximately, whereas the lower and upper limits under ideal conditions are between 110 Kbps to 290 Kbps, approximately.

**Figure 42:**
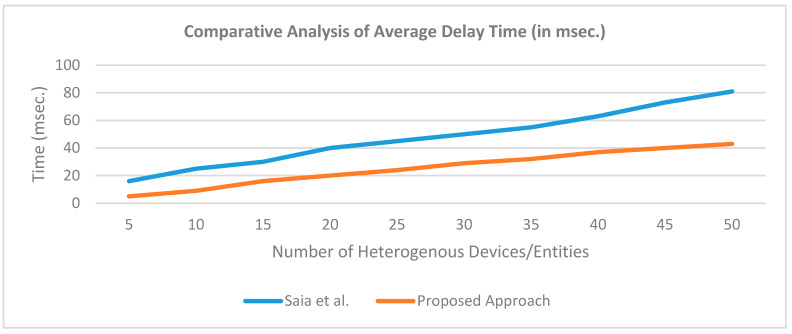
Comparative analysis of proposed work with existing using average delay time variations in consensus building.

Figure 42 shows the comparative analysis of the proposed work with the results of Saia et al. [19]. 

The results show that the delay in consensus building with different entities or devices in the network is lesser for the proposed approach as compared to the encryption/decryption-based approach used in Saia et al. [19]. This delay constantly increases with an increase in the number of devices. This experimentation is considered for 50 devices. However, the trends in results show that the delay gap increases. Thus, the proposed approach is much better compared tothe existing approach for small devices based networks to large device networks.

## 5. Conclusions

This work shows the advantages of a patient or data-centric system compared to specialization or department-centric practices in the healthcare domain. The patient or data-centric system advantages are analyzed with a wearable kidney-based simulation model. It has been observed in the simulation that blockchain technology, Industry 4.0 processes, and data-centric systems bring transparency, trust, and easy and fast accessibility. Thus, it would be easy to control, regulate and monitor artificial medical automated body parts (like wearable kidneys). Here, there would always be an option to monitor and control these medical body parts by a doctor or patient itself. Further, taking resource-constrained electric devices (in artificial medical automated body parts) into consideration, a blockchain technology-based PoG consensus algorithms are proposed to have a faster, secure, and trustworthy data-centric network. In this work, Healthcare 4.0 processes (having Industry 4.0 processes capability) and blockchain technology-based simulation models are proposed specifically for the kidney system. The simulation shows the working of a normal kidney, wearable kidney, and their integration with proposed Healthcare 4.0 and blockchain-based processes. In results, average doctor utilization is found to be close to 98% and network ADPR lies between 95% to 97% approximately without the presence of outliers. In the presence of outliers, network performance decreases below 80% APDR (to a minimum of 41.3% in 10-simulation experimentation) and this indicates that there are outliers present in the network. Simulation results show that the AT value lies between 120 Kbps to 250 Kbps for the proposed network. This study and experimentation show that there are numerous advantages of the proposed system in electronic medical records, supply systems, biomedical research, and education, data analytics etc. Thus, many prototypes based on blockchain processes (having decentralized networks, smart contracts, on or off-chains, trust-building system etc.) can be designed in the future for healthcare sector advancements. Further, there is a need to conduct more theoretical or experimental research to evaluate the integration of blockchain technology in Healthcare 4.0 processes keeping their challenges (like interoperability, privacy, scalability, performance, QoS, etc.) into consideration.

## Figures and Tables

**Table 1:** Comparative state-of-the-art survey of consensus algorithms in various blockchain-based healthcare applications.

Author	Year	Consensus Algorithm & Salient Features
*Proof-of-Work, Proof-of-Stake, Proof-of-Trust, Proof-of-Concept*		
Witchey [21]	2015	This work has mainly discussed the proof-of-work consensus algorithm for healthcare transactions. The healthcare data is provided to one or more validation devices. Thus, the consensus of these devices is maintained through the proof-of-work concept. This work has explored the various other consensus approaches discussed and developed in blockchain technology. However, proof-of-the work is mainly suggested for healthcare applications.
*Proof-of-Concept, Proof-of-Stake, Proof-of-Insurance*		
Nichol et al. [22]	2016	This work has mainly discussed proof-of-concept importance in building the trust of the healthcare system with associated applications. These applications are mainly in the healthcare field that requires interoperability. This work has discussed the blockchain healthcare ecosystem suitable for patient or data-centric approach underlying theoretical framework and conceptualized proposition. This need is added to have patient satisfaction, trust in the healthcare domain, removing frauds, security risks reductions etc.
*Proof-of-Work, Proof-of-Interoperability*		
Peterson et al. [2]	2016	This work has proposed proof of structural and semantic interoperability and mainly used proof-of-work in sharing healthcare data. This work has concentrated over applying consensus algorithms in institutions interoperability rather than concentration over the patient or data-centric issues. This work has mainly concentrated over sharing the patient’s and healthcare-related data with interested and trusted parties while keeping data quality and utility intact. Further, patients and the institution’s concerns are discussed to have an aggregated blockchain with machine learning capabilities.
Zhang et al. [23]	2016	This work added the cryptography mechanisms to blockchain creation in healthcare. Elliptic curve cryptography and its variations are used for ensuring security. Further, a formal verification model is created to analyze the proposed approach. Results show that the proposed approach is protected from various types of attacks. Variations in elliptic curves show that the average runtime of the proposed approach increases with an increase in complexity. Here, a blockchain network is constructed with the help of wearable sensor devices. These devices exchange information through mobile gateways which further records the transactions in the blockchain network. Each transmitter/receiver is made available to every blockchain node that collected the data through wireless wearable sensors.
Alhadhrami et al. [13]	2017	This work has integrated blockchain with the healthcare system and proposed a model to share medical and healthcare records among patients, doctors, hospitals, nurses etc. The aim of sharing this information is to increase interoperability among various sub-systems. Here, a framework is only proposed to have the blockchain integrated functionalities and advanced security levels with verifications from every stakeholder in the network. In another observation, the chances of blockchain failure with quantum computers are discussed theoretically in addition to chances of attacks (such as double proof, blackhole, Sybil attack).
Zhang et al. [24]	2017	This work has mainly addressed the proof-of-interoperability and its importance in the healthcare domain. This is a theoretical constitution to address, explore and analyze the present and future need of blockchain-based healthcare systems. Here, a DApp for Smart Health (DSH) framework is proposed as well to maintain the evolvability with minimum integration complexity. This work has realized that interoperability is the major concern in present healthcare scenarios. Thus, a balancing approach is required to enhance the data availability is based on interoperability rather than any other form of integration. This interoperability can be achieved efficiently through proper security measures. The proposed framework is good in terms of providing data to research, innovation, and analysis. These technicalities would help anyone to understand the relevant health changes across the targeted population and monitor them in an efficient way.
Zhang et al. [25]	2017	This work is an extension of work done to integrate interoperability in the healthcare sector [11]. Interoperability through data is preferred to have a patent centric system. A transparent system to both doctor and patient will bring trust and encourage to have healthy practices and remove frauds. This is possible through blockchain-based solutions. Thus, a DASH architecture is proposed and explained in this work. The complexity of the system is considered by designing various activities using UML diagrams. The detailed functionality of each sub-system is explained with exceptions through designs only. There is a further need to prove the proposed designs with statistical and implementations methods.
*Proof-of-Concept*		
Engelhardt [26]	2017	This work has observed that the integration of blockchain technology would bring various advantages to the healthcare system with better administration, monitoring, and accessibility. These claims are made with concrete examples having clear and specified near and long-term goals, promises, and challenges. This theoretical framework based discussion is fruitful to have an understanding of a pre-specified set of stakeholders, their roles, regulations, and other technical details. It is observed that academic discussions are not just helpful for addressing the healthcare needs but to explore various other healthcare-associated system and their interoperability in the blockchain-based modern architecture.
*Proof-of-Work, Proof-of-Stake, Proof-of-Burn*		
Kuo et al. [27]	2017	This work has introduced the Bitcoin-based blockchain technology with proof-of-work in a decentralized mechanism-based healthcare system. The major topics discussed in the proposed healthcare framework are security, availability, robustness, data immutability etc. The discussed framework mainly explores the feasibility of blockchain in biomedical and healthcare applications, research or record management. Here, the blockchain-based double-spending and single-point-of-failure are discussed with healthcare blockchain scenarios. Additionally, the security measures to enhance the blockchain technology are addressed as well. Moreover, the presented work is theoretical rather than practical or statistical proving approach.
*Proof-of-Existence, Proof-of-Verification, Proof-of-membership*		
Xia et al. [28]	2017	This work has concentrated over interoperability through data sharing in a blockchain and cloud environment based healthcare system. This healthcare system emphasizes medical records over institution interoperability. Thus, a multilayered approach is proposed to have three consensus algorithms operated through cryptography primitives and protocols. The proposed system has user, system management and storage layers. The user layer defines the different types of users that can interact with the system to have data accessibility and interactions. The system management layer defines the roles of the issuer, verifier, consensus nodes, pool of transactions and blockchain technology-based network construction. Finally, the storage layer defines various types of storage and classification of data. Each of these layers does not have a role without blockchain and cloud computing features and functionalities.
*Cryptography and game-theory*		
Zhang et al. [29]	2017	This work has realized that the present system does not interconnect the heterogeneous data sources to have interoperability whereas the proposed decentralized application will connect them to have a better environment. The healthcare environment would be better in terms of data accessibility and fault tolerance approaches. This work has explored the blockchain expectations, scalability issues in the present and blockchain-based healthcare system, cost-effectiveness, patient-centered support etc. Here, technical evaluations regarding these issues are explored theoretically and with existing Ethereum based architecture.
*Proof-of-Work*		
Funk et al. [30]	2018	This work has realized the easiest implementation of blockchain in healthcare professional education. Here, the details of the regulatory body, technology, competency matrix, online learning, online courses, and media-based education are discussed for exploring and finding the trends of blockchain application in the real-time healthcare sector. This work is a short survey of the possibility of blockchain implementation in healthcare education and professionals. The platform expectancy is to develop efficient information sharing, structured, verifiable, trusted, accountable, incentivized and secure platforms for healthcare education.
Gordon et al. [31]	2018	This work emphasized over patient-centric data sharing and management system compared to the institution or specialization-centric system. Thereafter, the barriers to patient driving interoperability are explored, analyzed and facilitated for the transition from the present system to a patient-centric approach. This article specifies the interoperability feature comparison of two systems: (i) the present hospital, clinics, pharmacies, doctors-based present system, and (ii) blockchain-based data-centric system for electronic health and medical records.
Mamoshina et al. [32]	2018	This work hs presented the details of artificial intelligence and blockchain technology-based innovative healthcare solutions that can be used to speed-up the research aspects in the biomedical field. Further, the patient will be able to appraise and evaluate his/her records in a way that he/she chooses to be the best. In this work, various designs are proposed to have service instance, auditing, authenticity and load balancing in blockchain-based healthcare applications. Further, deep neural networks are applied and trained to find the experimental results from the patient data and its feature extraction processes.
Zheng et al. [33]	2018	This work has proposed conceptual design for data sharing in a secure and transparent manner with blockchain, cloud computing, and machine learning approaches. The goal of this work is to enroll users to self-control their data, apply security approaches in sharing data with associated stakeholders in the healthcare system under the General Data Protection Regulation (GDPR) compliance.? The proposed architecture applies data quality check at its initial steps, integrated necessary security primitives and protocols with pre-processed and formatted data having recorded with timestamps, cloud storage enables to store data in encrypted form, and accessibility to users and consumers through proper application interfaces.
*Proof-of-Work, Proof-of-Concept*		
Griggs et al. [34]	2018	This work has implemented a private blockchain using Ethereum protocol. Here, sensors are used to communicate with smart devices and provide data for all associated events on the blockchain. This data is more trustworthy because everyone is bound with smart contracts that are automated. In an example, sensors are associated with the human body and a master device (like mobile) receives the data from these sensors and transmits to the blockchain network. In parallel, a smart contract executes that look after the compliances of any data related issue.
*Multiple (Proof-of-work, Proof-of-Stake, Proof-of-Activity, Proof-of-Burn, Proof-of-Deposit etc.)*		
Hölbl et al. [35]	2018	This is a review work for analyzing the contributions in the field of blockchain technology usage in the healthcare domain. There are various statistics shown in this work to present the need and advantages of blockchain technology through several publications and findings. This work has discussed multiple distributed consensus protocols such as proof-of-work, proof-of-stake, delegated-proof-of-stake, proof-of-importance, proof-of-activity, proof-of-burn, and proof-of-deposit etc. These distributed consensus algorithms are presented w.r.t the present stage of their usage. Additionally, various advantages, applicability and other technical details about blockchain are presented in this work.
*Proof-of-Stake*		
Shen et al. [36]	2019	In this work, an efficient data-sharing scheme is proposed for the healthcare system using blockchain technology named as MedChain. The proposed scheme uses a session-based healthcare data sharing approach for finding the flexibility in the data availability approach securely and efficiently. Medchain architecture considers every stakeholder as a node in a peer-to-peer architecture. In this architecture, two types of services run (i) directory and (ii) blockchain. Directory services provide database accessibility whereas, blockchain services ensure security, transparency, immutability and consensus-building advantages.
*Proof-of-Work, Proof-of-Stake, Proof-of-Byzantine Fault Tolerance*		
De Aguiar et al. [37]	2020	This work has surveyed over the various approaches applied in integrating the blockchain technology in the healthcare sector. Various types of data such as medical records, image sharing, and log management are found to be important in the healthcare system. This survey is advanced to others in terms of detailed benefits and limitations of healthcare, IoT and blockchain integration with healthcare, proposing techniques that are found to be useful in the healthcare domain, data access control feature system with improved health records and how it helps in improving the statistics, use cases for monitoring and remote accessibility etc.
